# Internet fraud transaction detection based on temporal-aware heterogeneous graph oversampling and attention fusion network

**DOI:** 10.1371/journal.pone.0337208

**Published:** 2025-12-05

**Authors:** Sizheng Wei, Suan Lee

**Affiliations:** 1 School of Finance, Xuzhou University of Technology, Xuzhou, Jiangsu, China; 2 School of Computer Science, Semyung University, Jecheon, Republic of Korea; Philadelphia University, JORDAN

## Abstract

This study proposes an advanced Internet fraud transaction detection method, the Temporal-aware Heterogeneous Graph Oversampling and Attention Fusion Network (THG-OAFN), designed to address the increasingly severe fraud issues in EC. The method innovatively abstracts transaction data into a heterogeneous graph structure, captures temporal dynamic features through Gated Recurrent Unit (GRU), and fuses Graph Neural Network (GNN) to process static topological relationships. To address data imbalance, an improved Graph-based Synthetic Minority Oversampling Technique (GraphSMOTE) framework is introduced, maintaining the structural integrity of fraud clusters through k-hop topological constraints. Meanwhile, a multi-layer attention mechanism (including relationship fusion, neighborhood fusion, and information perception modules) is employed to achieve active fraud prevention. Experimental results show that THG-OAFN attains an area under the curve (AUC) of 96.56% (a 7.78% improvement over the best baseline). Moreover, it achieves a recall of 95.21% (a 6.29% improvement) and an F1-score of 94.72% (a 3.96% improvement) on the Amazon dataset. On the YelpChi dataset, these three metrics reach 90.43%, 89.51%, and 90.31%, respectively, remarkably outperforming existing GNN models. This achievement provides a deployable solution for dynamic fraud detection and active defense. Our code is available at https://github.com/wei4zheng/THG-OAFN.

## 1. Introduction

With the booming development of E-commerce (EC), Internet fraud transactions have become an increasingly serious problem, causing significant economic losses to both individual users and businesses [1]. Firstly, fraudulent transactions result in direct economic losses, including refunds, compensations, and legal fees, while also increasing business transaction costs. Secondly, fraudulent activities undermine consumers’ trust in EC platforms, potentially leading to user attrition and affecting businesses’ market share and revenue. Additionally, businesses must comply with various laws and regulations to protect consumers’ financial information and privacy, and fraudulent transactions may expose businesses to litigation and regulatory penalties, increasing compliance costs. Fraudsters continuously adopt new technologies and strategies to evade detection, forcing businesses to continually update their fraud detection systems to counter evolving fraudulent methods. Furthermore, the high dimensionality, large scale, and rapid growth of Internet transaction data make it increasingly challenging to accurately identify fraudulent behaviors from massive amounts of data [2–5]. Therefore, developing a fraud detection method that can simultaneously consider the timing and dynamics of transaction data as well as the evolution pattern of fraudulent behavior is of great significance for improving the accuracy of detection and prevention measures.

Traditional fraud detection methods have three fundamental limitations. First, regarding temporal dynamic capture, rule engines and static machine learning models cannot model the evolution of transaction frequency and amount (such as the “low-frequency testing-high-frequency outbreak” periodic pattern commonly used by fraud rings). This leads to delayed responses to implicit early risk signals in transaction sequences (such as consecutive small exploratory transactions) [6–8]. Second, when facing extremely imbalanced data, traditional Synthetic Minority Oversampling Technique (SMOTE) can disrupt topological integrity when generating synthetic nodes in graph structures. For example, random interpolation leads to the fragmentation of the community structure of the fraud cluster. Meanwhile, existing methods fail to address the problem of attribute completion error propagation [9]. Third, for real-time performance, manual rule-based systems have update cycles as long as weeks, unable to adapt to high-frequency transaction scenarios with tens of thousands of transactions per second. In contrast, batch-processing machine learning models struggle to meet the sub-hundred-millisecond response requirement [10]. These shortcomings collectively result in existing systems achieving a detection rate of less than 35% in dynamic financial environments. Moreover, previous studies have not addressed the collaborative optimization problem of data imbalance and active prevention.

Therefore, it is of great significance to develop a fraud detection method that can simultaneously consider the timing and dynamics of transaction data as well as the evolution pattern of fraudulent behavior to improve the accuracy of detection and prevention initiatives. This study aims to propose an advanced fraud detection method, Temporal-aware Heterogeneous Graph Oversampling and Attention Fusion Network (THG-OAFN), to address the increasingly severe issue of fraud in EC. The THG-OAFN model abstracts transaction data into a heterogeneous graph, utilizes a Gated Recurrent Unit (GRU) to capture temporal data, and combines a Graph Neural Network (GNN) to process static features.

Passive detection relies on historical rule matching for identified fraud patterns (such as blocking blacklisted accounts), essentially functioning as a “post-event response” strategy. In contrast, active prevention predicts potential risks through a multi-layer attention framework to achieve “pre-event interception.” The core value of active mechanisms in temporal financial data is as follows. When fraudulent behaviors are still in the early stages (e.g., the quota tentative period of a stolen account), the model can capture weak abnormal association signals through the relationship fusion module, providing earlier warnings than passive detection. This early intervention capability holds irreplaceable defensive value for minimizing fraud losses, particularly for “latent” financial crimes (such as the slow fund aggregation in money laundering networks).

The four innovations of this study directly point to the core gaps of existing research:

(1) Temporal-sensitive modeling: Existing GNN-based fraud detection models rely on static graph snapshots, ignoring the temporal evolution of transaction behaviors. The GRU-GNN coupled architecture of THG-OAFN achieves joint modeling of transaction frequency fluctuations and topological evolution for the first time, addressing the “concept drift” problem (e.g., monthly iterations of fraud strategies).
(2) Topology-preserving oversampling: This study proposes k-hop similarity constraints and a dual-channel decoder to address the topological disruption issues in methods like Graph-based SMOTE (GraphSMOTE). These innovations specifically enhance the structural similarity between synthetically generated nodes and original fraud clusters.
(3) Active prediction mechanism: Existing studies still belong to the passive response paradigm. This study realizes active sniffing of potential risk patterns through a three-layer attention framework (relationship/neighborhood/information), improving the early fraud detection rate of passive models.
(4) The dynamic adaptation system: It breaks through the limitations of static models by designing an incremental learning-knowledge base feedback loop, enabling the model to maintain a high, stable F1-score in high-frequency data streams.

## 2. Literature review

### 2.1 Traditional fraud detection technology

Internet fraud detection is a vital branch of the information security field. In recent years, with the prevalence of online transactions, research in this field has received widespread attention. Zheng et al. proposed an improved transfer AdaBoost algorithm for handling scenarios where data distributions changed slowly over time. Especially in credit card transaction data, this change could lead to concept drift issues, which were crucial for detecting transaction fraud [11]. Zhang et al. introduced a network attack detection method combining flow calculation and deep learning (DL) [12]. Their methods and findings in real-time data processing, application of DL techniques, and experimental verification offered valuable references and insights for research in the Internet fraud transaction detection domain. Ashfaq et al. studied fraud and anomaly detection issues in the Bitcoin network [13], using XGBoost and Random Forest (RF) algorithms for transaction classification and pattern prediction. Although these methods have improved detection accuracy to some extent, three main deficiencies remain. First, they struggle to capture the temporal dependency features in transaction behaviors and cannot effectively characterize the periodicity and temporal evolution of fraud activities. Second, traditional oversampling methods like SMOTE cannot handle graph structures, easily introducing topological disturbances during node generation. Third, rule engines rely on manual maintenance and updates, making them difficult to adapt to high-frequency transactions and cross-platform data scenarios. Therefore, although such methods are more suitable for application environments with stable structures and single fraud types, they become inadequate when facing dynamically evolving fraud patterns.

### 2.2 A fraud detection method based on static and heterogeneous graphs

Heterogeneous GNN has become a hot research topic in fraud detection because of its advantages in processing graph data with rich types of nodes and edges. For example, Li et al. presented a graph learning algorithm, TA- Topological and Attribute Structure to Vector (Struc2Vec), for internet finance fraud detection [14]. This algorithm could learn the topological and transaction amount features in financial transaction network graphs, representing them as low-dimensional dense vectors. These vectors were then used for intelligent and efficient classification and prediction by training classifier models. Tan et al. constructed transaction graphs through the Ethereum network and proposed an embedded behavior modeling method to classify address types [15]. Arfeen et al. proposed an ML-based multilayer framework for detecting and classifying abnormal behaviors in online financial transactions [16]. The framework could help financial service providers avoid events such as network intrusions and online fraud, and enhance the credibility of online financial platforms or gateways. Megdad et al. evaluated the effectiveness and efficiency of different ML algorithms, including the Naive Bayes classifier, AdaBoost classifier, decision tree, K-nearest neighbor classifier, logistic regression, and Bagging classifier, in detecting risks in financial transactions. Their study aimed to improve customer experience and minimize financial losses [17]. These methods have enhanced the expressive ability for complex graph structures. However, their core limitation lies in the fact that the graph structure is static and cannot model the temporal evolution process. The attributes of nodes and edges are usually fixed, making it difficult to capture the changing trends of transaction behaviors. More importantly, static graph methods have learning biases when dealing with extremely unbalanced data distributions and are often insensitive to the identification of minority classes (i.e., fraudulent behaviors). Moreover, the introduction of oversampling in the graph structure often leads to the disorder of topological relations. For instance, SMOTE generates false neighbors on attribute-free nodes, which may disrupt the clustering structure of the graph. To address these issues, Wen et al. (2024) proposed the Graph Tran-SMOTE method, which combined graph embedding and oversampling strategies to effectively enhance the fraud detection ability in imbalanced graph data [18].

### 2.3 Dynamic graph fraud detection method considering temporal characteristics

To capture the dynamic change characteristics of fraudulent behaviors more comprehensively, research has begun to introduce dynamic graph modeling and time-aware mechanisms in recent years. This type of method emphasizes the modeling of the transaction occurrence sequence, timestamps, and behavioral evolution paths, and is suitable for high-frequency transactions and fraud detection scenarios with complex behavioral evolution. For instance, Tan et al. detected fraudulent transactions by mining Ethereum transaction records [19]. This framework included using web crawlers to obtain Ethereum addresses labeled as fraudulent/legal, thus constructing transaction networks. Moreover, they proposed a transaction behavior-based network embedding algorithm to extract node features and used a Graph Convolutional Neural (GCN) model for address classification. Long et al. demonstrated a hybrid GNN model to handle three types of imbalances in online transaction fraud detection: feature imbalance, class imbalance, and relationship imbalance [20]. Innan et al. proposed a new method using Quantum Graph Neural Networks (QGNN) for financial fraud detection [21]. The experimental results revealed that QGNN achieved an Area Under the Curve (AUC) of 0.85, outperforming traditional GNN, demonstrating the potential of QGNN in fraud detection tasks. Ren et al. further proposed a dynamic graph method based on imbalanced structure learning, effectively integrating the graph topological structure and the class skew distribution [22]. Furthermore, the GAT-COBO model developed by Hu et al. was cost-sensitive and suitable for dynamic graph analysis in scenarios such as communication fraud [23]. Khosravi et al. proposed a transaction fraud detection method based on attention spatiotemporal GNN [24]. This method dynamically captured spatial correlations and temporal evolution features in transaction data through an attention mechanism. It focused on exploring the indicative role of node interaction patterns in fraud behavior under spatiotemporal dimensions, providing new ideas for pattern mining in dynamic transaction scenarios. Rahmati focused on real-time financial fraud detection and proposed a framework combining adaptive GNN and federated learning [25]. Its core realized privacy-preserving training of cross-platform data through a federated learning architecture. Meanwhile, it used an adaptive GNN to dynamically adjust model parameters to adapt to high-frequency transaction environments, offering a feasible path to resolve the conflict between real-time performance and data privacy. Takahashi et al. developed a GNN model for financial fraud prevention [26], with the basic graph structure of financial transaction networks as the core. By extracting topological correlation features between nodes through GNN, the model strengthened the ability to capture potential fraud signals in basic transaction relationships. At the same time, it provided a reference for designing basic models for fraud prevention in the financial field. Pérez-Cano et al. explored the combination of anomaly detection and heterogeneous graph transformers for fraud detection in cryptocurrency networks [27]. This study concentrated on complex networks composed of multiple types of nodes and edges in cryptocurrency transactions, and modeled heterogeneous relationships through heterogeneous graph transformers; it also identified transaction behaviors deviating from normal patterns by combining anomaly detection algorithms, expanding the application of heterogeneous graph models in specific financial scenarios. Jiang et al. proposed a Multi-View Contrastive Graph Neural Network (MVCG-SPS) for intelligent Ponzi scheme detection [28]. This method extracted transaction network features from different dimensions through multi-view learning and enhanced the feature discriminability using contrastive learning. It focused on solving the feature representation problem of complex fraud patterns such as Ponzi schemes, offering a new perspective for fraud detection with multi-source information fusion. These methods have made breakthroughs in time series modeling and data imbalance management, but they still face two challenges. First, most methods only rely on a single-layer attention mechanism, which makes it difficult to achieve high-level information fusion. Second, the update strategy of dynamic graphs is based on full reconstruction, lacking an efficient incremental learning mechanism and unable to adapt to the rapid evolution of fraudulent behaviors promptly.

### 2.4 Summary and problem location

The summary of the advantages and disadvantages of the work related to Internet fraud transaction detection is exhibited in Table 1. In summary, current fraud detection methods have evolved from rule-driven to structural modeling and then to dynamic graph integration, but key challenges remain unsolved. Traditional methods have limited capability to represent time series and high-dimensional unstructured features. Static graph approaches ignore temporal dependencies and exhibit poor robustness under imbalanced data conditions. While dynamic graph methods incorporate temporal factors, they remain incomplete in information fusion, structural integrity preservation, and low-resource learning. Notably, the application of oversampling in graph structures still suffers from topological perturbation issues, leading to model misidentification of edges or nodes. More critically, most current mainstream methods are “passive detection”. For example, they only identify behaviors after they occur, lacking future-oriented active prediction and risk perception mechanisms.

**Table 1:** Summary of the advantages and disadvantages of the relevant work on Internet fraud transaction detection.

Literature and methods	Advantages	Disadvantages
Zheng et al. [11]	An improved transfer AdaBoost algorithm adapted to concept drift and improved detection accuracy	The temporal dependence of trading behavior has not been fully captured, making it difficult to handle complex temporal characteristics
Zhang et al. [12]	Combined flow calculation and DL, supported real-time data processing	The traditional DL model has limited ability to express heterogeneous relationships and graph structures
Ashfaq et al. [13]	By using XGBoost and RF classification comparison, the accuracy of anomaly detection for Bitcoin transactions could be improved	They ignored the structural features of the graph and were sensitive to data imbalance, making it difficult to maintain structural integrity
Li et al. [14]	Heterogeneous graph learning based on Struc2Vec could extract low-dimensional representations of topological and transaction features	The graph structure was static, and the attributes of nodes and edges were fixed, lacking the ability to model the evolution of behavior
Tan et al. [15]	Constructed transaction graphs through the Ethereum network and proposed an embedded behavior modeling method to classify address types	The static graph method responded insufficiently to dynamically changing fraud patterns
Arfeen et al. [16]	A multilayer ML framework effectively detected abnormal transaction behaviors	Lacked a temporal dynamic analysis, making it difficult to capture complex behavioral evolution paths
Megdad et al. [17]	They systematically evaluated the performance of multiple traditional ML algorithms, focusing on customer experience and financial risks	Traditional algorithms exhibited a weak perception of graph structures and were difficult to handle large-scale and complex graph data
Wen et al. [18]	Graph Tran-SMOTE combined graph embedding and oversampling strategies to enhance the fraud detection ability of imbalanced graph data	Their work was still based on static graphs, and the topological structure disturbance caused by oversampling had not been completely avoided
Tan et al. [19]	A dynamic graph approach that combined Ethereum transaction sequences and timestamps to model the behavioral evolution path	The model had high complexity, lacked a multi-layer attention mechanism integration, and the real-time update mechanism was insufficient
Long et al. [20]	A hybrid GNN to handle three types of imbalances: feature, class, and relationship	It mainly relied on single-layer attention, and the depth of information fusion was limited
Innan et al. [21]	QGNN enhanced AUC performance, demonstrating the potential to drive new types of financial fraud detection	The implementation of quantum models was difficult, and their practical applications were limited
Ren et al. [22]	Dynamic graph structure learning, combined with class skew, could enhance the modeling effect of imbalanced data	The update strategy was based on full-graph reconstruction, which had a relatively slow response speed and could not quickly adapt to the rapid changes in fraudulent behavior
Hu et al. [23]	The cost-sensitive GAT-COBO model was suitable for dynamic graph analysis of communication fraud	The attention mechanism was single-layered, and the information fusion was insufficient
Khosravi et al. [24]	Attention spatiotemporal GNN captured the spatiotemporal features of transactions	The attention mechanism was single and failed to address the issues of data imbalance and topological integrity
Rahmati [25]	Adaptive GNN, combined with federated learning, could balance both privacy and real-time performance	Federated learning displayed low aggregation efficiency and insufficient response to extreme dynamic fraud
Takahashi et al. [26]	GNN captured fraud signals in the fundamental relationships of financial transactions	The lack of temporal dynamic features limited the expression of complex heterogeneous relationships
Pérez-Cano et al. [27]	Anomaly detection and heterogeneous graph converters were suitable for cryptocurrency networks	Limited to the cryptocurrency scenario, the issues of topological perturbation, and dynamic adaptation have not been resolved
Jiang et al. [28]	MVCG-SPS improved the discriminability of Ponzi scheme features	Lacked dynamic weight adjustment and time series modeling, making it weakly adaptable to data imbalance

The THG-OAFN model proposed in this study addresses these issues through an innovative design. The method enhances the ability to identify critical nodes from three dimensions (neighbor aggregation, relationship fusion, and information perception) via a multi-layer attention mechanism. It introduces temporal dependency features through a GRU-GNN joint structure to strengthen the modeling of transaction evolution patterns. A graph oversampling strategy based on embedding space is employed to alleviate class imbalance, while an incremental update mechanism enables continuous expansion of the model’s knowledge base. Unlike “passive detection,” THG-OAFN emphasizes “active prevention”. By learning the evolutionary paths of user historical behaviors, it predicts potential risks and intervenes in high-risk nodes in advance, significantly improving responsiveness to new types of fraudulent behaviors.

## 3. Method

### 3.1 Preliminaries and notation

In this subsection, we summarize the key terms and mathematical symbols used throughout the paper to ensure a consistent and unambiguous description of the proposed THG-OAFN framework. Table 2 presents the core terminology, including the overall architecture (THG-OAFN), fundamental concepts in heterogeneous graph modeling, and the main components such as GRU, GNN, SMOTE, and GraphSMOTE that are integrated into our method. Table 3 lists the symbols related to heterogeneous graph construction, attention-based feature aggregation, attribute completion, SMOTE-based node generation, and edge-aware relationship modeling, which are used in the formal definition of our model and training procedure. Unless otherwise specified, these definitions and notations are adopted consistently in the following sections.

**Table 2:** Term definition.

Term abbreviation/name	Full name (if applicable)	Function/definition description
THG-OAFN	Temporal-aware Heterogeneous Graph Oversampling and Attention Fusion Network	The overall detection framework proposed in this study integrates temporal perception, graph modeling, oversampling, and attention mechanisms
Heterogeneous Graph	–	A graph composed of multiple types of nodes and edges is used to simulate complex transaction behaviors and the relationships between entities
GRU	Gated Recurrent Unit	It is used to model transaction time series changes and capture long-term dependent time series characteristics
GNN	Graph Neural Network	It aggregates the neighbor node information in the graph to achieve static structure learning
SMOTE	Synthetic Minority Oversampling Technique	New samples are synthesized in the feature space to alleviate the problem of minority class imbalance
GraphSMOTE	Graph-based SMOTE	New nodes are generated on the basis of considering the graph structure topology to maintain structural consistency

**Table 3:** Symbol table.

Symbol	Meaning	Module
G=(V,E)	A heterogeneous graph; V is the set of nodes; E is the set of edges	Heterogeneous graph modeling
Q	A set of node types	Heterogeneous graph modeling
R	A set of edge types	Heterogeneous graph modeling
ϕ:V→Q	Node-type mapping function	Heterogeneous graph modeling
ψ:E→R	Edge-type mapping function	Heterogeneous graph modeling
N(n)	A set of neighborhood nodes for node n	Attention mechanism
$h_{n}$	Eigenvector of the node *n*	Feature extraction
$e_{ns}$	The attention score of node *n* and neighbor *s*	Attention mechanism
W	Learnable parameter matrix (Feature transformation)	Attention mechanism
a	Attention weight vector	Attention mechanism
LeakyReLU	Activate function with a default negative slope of 0.2	Attention mechanism
$\alpha_{ns}$	Normalized attention weights	Attention mechanism
K	The number of heads of the multi-head attention mechanism	Attention mechanism
$\hat{x}$	Refactored attribute vectors	Attribute completion
x	Raw attribute vectors	Attribute completion
$V_{m}$	A set of nodes with missing attributes	Attribute completion
$V_{c}$	A set of nodes with complete attributes	Attribute completion
δ	SMOTE interpolation coefficient, 0<δ<1	SMOTE generation
$h_{v}^{\text{1}}$	The first-layer embedding representation of node v	SMOTE generation
${\hat{h}}_{v^{\prime }}^{\text{1}}$	An embedding representation of the newly generated node	SMOTE generation
A	Original adjacency matrix	Graph structure modeling
$\tilde{A}$	The adjacency matrix after the new edges are generated	Graph structure update
$W_{r}$	Node relationship weight matrix (Edge-type awareness)	Relationship fusion
Decoder(u,v)	Relational scoring function based on inner product	Edge generation module
$L_{\text{completion}}$	Attribute refactoring loss function	Loss function
$L_{\text{edge}}$	Prediction loss function based on edge generation	Loss function
L	Overall joint loss function	Model training

### 3.2 The overall framework of the THG-OAFN model

**Fig 1:**
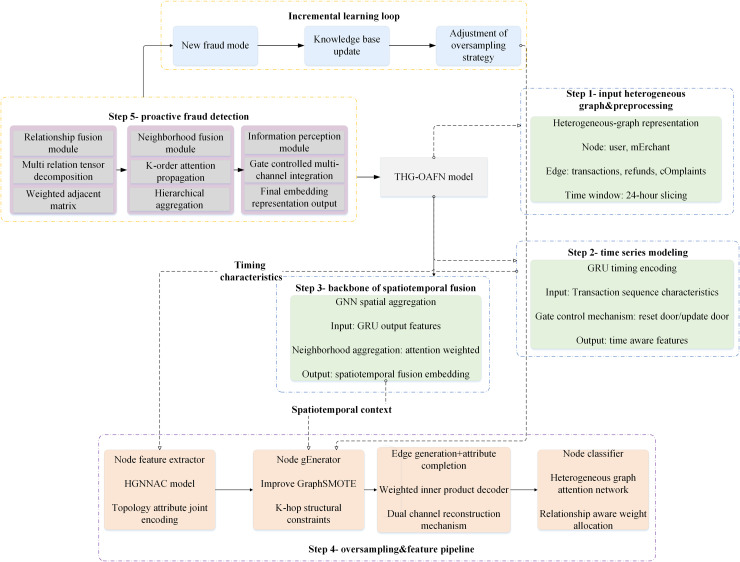
The overall framework of the THG-OAFN model.

The overall framework of the THG-OAFN model is a complex system that integrates time awareness, heterogeneous graph data abstraction, oversampling techniques, and attention mechanisms. This model enhances the accuracy and efficiency of fraud detection by comprehensively considering the temporal characteristics of transaction data, static features, complex relationships between nodes, and data imbalance issues. Fig 1 illustrates the THG-OAFN model’s overall framework, encompassing the temporal-aware heterogeneous graph oversampling and the active fraud detection modules. These two modules work in synergy to achieve dynamic fraud detection and active fraud prevention.

$\lambda_{\text{1}}H_{init}^{t}=FeatureExtractor(G^{t})H_{comp}^{t}=AttributeCompletion(H_{init}^{t})$ in Fig 1, the temporal-aware heterogeneous graph oversampling module first performs sliding-window segmentation on raw data based on transaction timestamps. Then, it converts transactions within each window into heterogeneous subgraphs containing multi-type nodes (users, merchants) and transaction edges. After the node initial representations are obtained via a feature extractor, the subgraphs enter the attribute completion and embedding-space SMOTE generation process. This process maintains local topological consistency while using Euclidean distance interpolation to generate minority-class nodes and corresponding candidate edges. Meanwhile, the process completes topological expansion through connection probabilities provided by an inner-product decoder. The generated new node features and updated adjacency matrices are written into a shared graph cache in real time, serving as inputs to the active fraud detection module.

The active fraud detection module receives dynamically expanding graph slices from the cache over time. It first fuses multi-type edges using learnable weight matrices at the relationship layer. Second, it calculates attention weights based on a LeakyReLU scoring function at the neighborhood layer to capture heterogeneous contributions of different adjacent nodes to target nodes. Node representations from multi-head attention and GRU hidden states within the time window are jointly fed into the information perception layer to generate final representations. They are then passed through a Heterogeneous Graph Attention Network classifier to output fraud probabilities. During module training, two types of losses are backpropagated. Attribute completion and edge prediction losses refine the oversampling module, while node classification losses update the detection module. Coordinated by a joint optimizer, the two modules form a closed-loop end-to-end collaboration. The pseudocode of the time-aware heterogeneous graph oversampling and active fraud detection algorithm is demonstrated in Algorithm 1.

**Algorithm 1:** THG-OAFN: Time-aware heterogeneous graph oversampling and active fraud detection algorithm.

**Input:**	Heterogeneous graph G, label set L, time window T, training epochs E
**Parameter:**	$\lambda_{\text{1}}$, $\lambda_{\text{2}}$,δ,τ,η: loss weight, oversampling ratio, attention dimension, learning rate, etc
**Output:**	The node embedding representation ${\left\{H^{t}\right\}}_{t=\text{1}}^{T}$, the classifier parameters Θ
1	Initialize the embedding $H^{\text{0}}$, the adjacency matrix $A^{\text{0}}$, and the model parameter Θ
2	**repeat**
3	**for** t=1 **to** T **do**
4	Construct the heterogeneous map slice $G^{t}$ in the tth time window
5	$H_{init}^{t}=FeatureExtractor(G^{t})$// Initial node feature extraction
6	$H_{comp}^{t}=AttributeCompletion(H_{init}^{t})$// Attribute completion
7	Generate a new minority node $V^{\prime }$ via SMOTE
8	A weighted inner product decoder is used to generate a new edge $E^{\prime }$: $\hat{A}=\sigma (H^{T}WH)$
9	Update graph structure: $G^{t}⟵G^{t}\cup (V^{\prime },E^{\prime })$
10	Calculation of attention mechanism:$a_{ij}=softmax(LeakyReLU(a^{T}\left[Wh_{i}|\left|Wh_{j}\right]\right))$
11	Neighbor information aggregation: $h_{i}^{\prime }=\sum_{j\in N(i)}\alpha_{ij}h_{j}$
12	Time update: $H^{t}=GRU(H^{t-\text{1}},H^{\prime })$
13	Fraud prediction: ${\hat{y}}^{t}=Classifier(H^{t})$
14	Calculation of the total loss: $L^{t}=L_{class}+\lambda_{\text{1}}L_{comp}+\lambda_{\text{2}}L_{dege}$
15	Parameter update: Minimize $L^{t}$ to optimize Θ
16	**end for**
17	**until** convergence
18	**return** ${\left\{H^{t}\right\}}_{t=\text{1}}^{T}$, Θ

### 3.3 Temporal-aware heterogeneous graph oversampling framework

**Fig 2:**
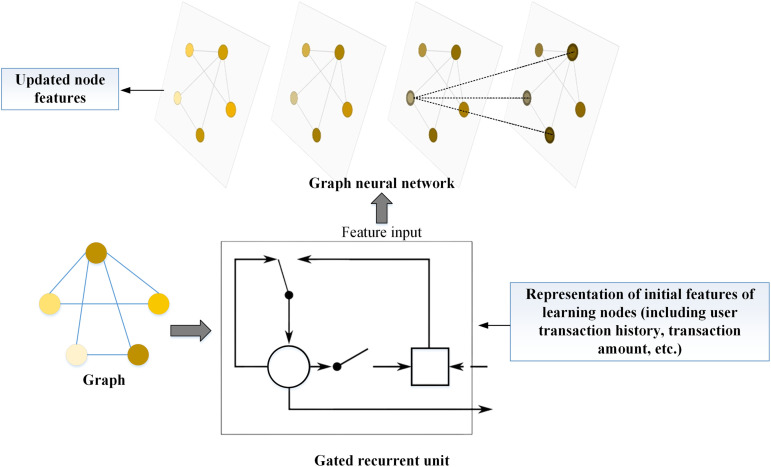
The integration of GRU and GNN.

The transaction data is abstracted as a heterogeneous graph, where nodes represent users and edges represent various types of transaction relationships between users. This abstraction enables the capture of complex interaction patterns, offering a rich data representation for fraud detection. To better understand and utilize the temporal information in transaction data, the GRU is tightly integrated with GNN. GRU, a variant of recurrent neural networks, is adept at capturing long-term dependencies and efficiently processing time series data through its gating mechanisms [29–31]. The integration of GRU and GNN is illustrated in Fig 2:

In Fig 2, the integration scheme of GRU and GNN aims to fully leverage the dynamic characteristics of time-series data and the static relationships of graph-structured data. First, GRU is employed to capture time-series features. Through its “update gate” and “reset gate” mechanisms, GRU updates the hidden state $h_{t}$ at each time step *t*, combining past information with curren*t* inputs to capture temporal dependencies in the data. Specifically, the computation for GRU is as follows:


$$r_{t}=\sigma (W_{r}x_{t}+U_{r}h_{t-\text{1}})$$ (1)

$$z_{t}=\sigma (W_{z}x_{t}+U_{z}h_{t-\text{1}})$$ (2)

$${\tilde{h}}_{t}=tanh(W_{h}x_{t}+U_{h}\left(r_{t}⊙h_{t-\text{1}}\right))$$ (3)

$$h_{t}=\left(\text{1}-z_{t}\right)⊙h_{t-\text{1}}+z_{t}⊙{\tilde{h}}_{t}$$ (4)

Eq. (1) calculates the reset gate, Eq. (2) calculates the update gate, Eq. (3) computes the candidate hidden state, and Eq. (4) updates the hidden state. Here, $x_{t}$ refers to the input at time step t; $h_{t-\text{1}}$ stands for the hidden state from the previous time step; W and U represent learned parameter matrices; σ denotes the sigmoid activation function; ⊙ means element-wise multiplication. Subsequently, GNN is used to leverage graph structure information for feature aggregation. GNN updates the feature representation of each node by propagating node features and incorporating information from adjacent nodes. In particular, graph convolution operations perform weighted aggregation of node features through the adjacency matrix *A*, and the updated node feature $h_{t}$ can be expressed as Eq. (5):


$$h_{t}=GNN\left(h_{t-\text{1}},A\right)=\sigma (\text{W}\cdot \sum_{i\in N(v)}A_{ij}h_{i})$$ (5)

$A_{ij}$ represents the edge weight between nodes i and j; N(v) denotes the set of neighboring nodes of node v; W refers to a learnable weight matrix; σ is an activation function. Through graph convolution operations, GNN can effectively aggregate information from neighboring nodes, enhancing the understanding of static graph structures.

To fuse the features from GRU and GNN, both concatenation and weighted averaging methods are adopted. The concatenation method obtains a joint feature representation by connecting the output features of GRU and GNN, which is then used for classification prediction through a fully connected layer. The weighted averaging method generates a weighted feature vector by assigning different weights to the outputs of GRU and GNN. Assuming the GRU’s output is $h_{GRU}$, and the GNN’s output is $h_{GNN}$, the computational process for weighted averaging fusion reads:


$$h_{fused}=\alpha \cdot h_{GRU}+(\text{1}-\alpha )\cdot h_{GNN}$$ (6)

α is a learnable weight parameter used to control the relative importance of GRU and GNN outputs. In this way, the model can dynamically adjust the fusion ratio of temporal features and graph structure features to adapt to different task requirements. The pseudocode for the GRU-GNN integration and fusion process is presented in Algorithm 2.

**Algorithm 2:** The GRU-GNN integration and fusion process.

**Input:**	Graph sequence ${\left\{G^{t}=(V^{t},A^{t})\right\}}_{t=\text{1}}^{T}$, Node label set L, training epoch E
**Parameter:**	Learning rate η, fusion weight α∈[0,1], GRU parameter $\Theta_{GRU}$, GNN parameter $\Theta_{GNN}$, classifier parameter $\Theta_{CLS}$
**Output:**	Predicted result ${\left\{{\hat{y}}^{t}\right\}}_{t=\text{1}}^{T}$; updated $\Theta_{GRU}$, $\Theta_{GNN}$, $\Theta_{CLS}$
1	Initialize GRU hidden state $h^{\text{0}}$, model parameters $\Theta_{GRU}$, $\Theta_{GNN}$, $\Theta_{CLS}$
2	**repeat**// Training epochs
3	**for** t=1 **to** T **do**
4	$X^{t}\leftarrow FeatExtract(G^{t})$// Extract the original features of the nodes
5	$h_{GRU}^{t}=GRU(X^{t},h^{t-\text{1}};\Theta_{GRU})$
6	$h_{GNN}^{t}=GNN(h_{GRU}^{t},A^{t};\Theta_{GNN})$
7	// **Fusion: Concatenation or weighted average**
8	${h^{t}}_{fused}=\alpha \cdot {h^{t}}_{GRU}+(\text{1}-\alpha )\cdot {h^{t}}_{GNN}$
9	${\hat{y}}^{t}=Classifier({h^{t}}_{fused};\Theta_{CLS})$
10	Calculate loss $L^{t}=CE({\hat{y}}^{t},y^{t})$
11	Backpropagation and update $\Theta_{GRU}$, $\Theta_{GNN}$, $\Theta_{CLS}$ (learning rate η)
12	**end for**
13	**until** convergence or until E epochs are reached
14	**return** ${\left\{{\hat{y}}^{t}\right\}}_{t=\text{1}}^{T}$, the updated model parameters

**Fig 3:**
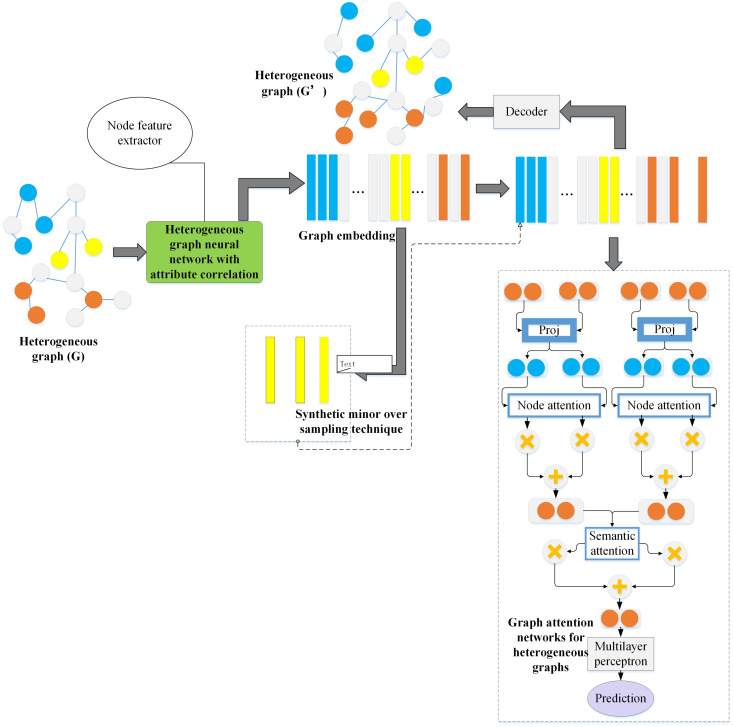
The structure of the heterogeneous graph-based oversampling model.

In fraud detection, data imbalance is a common problem. In other words, the data volume for normal transactions is often much larger than the amount of data for fraudulent transactions. A framework based on oversampling is proposed to solve this problem, which balances the ratio between normal users and fraudsters by generating new nodes [32]. The structure of the oversampling model based on the heterogeneous graph is presented in Fig 3:

Fig 3 illustrates that the node feature extractor employs the Heterogeneous Graph Neural Network with Attribute Correlation (HGNNAC) model. The orange bars in the figure represent the final prediction result or node feature, which is output through the multilayer perceptron. The newly generated nodes and edges significantly enhance the representation of a few class nodes, thus helping to improve the fraud detection performance of the model. This model can learn multiple feature representations of nodes from heterogeneous graphs, involving users’ transaction behavior, amount, frequency, etc., and offer rich feature information for subsequent node generation and classification [33]. The advantage lies in the HGNNAC model’s ability to learn complex feature representations across diverse types of nodes and edges. For instance, it can simultaneously consider users’ social relationships, transaction behaviors, and other critical attributes, thus improving its ability to detect fraudulent transactions. By generating new fraud nodes, the model enhances its ability to capture fraudulent behavior in the case of imbalanced data. These generated nodes increase the quantity of fraud data and help improve the model’s precision and recall in fraud detection. Considering the variability of fraudulent behavior, the model can rapidly adapt and learn new fraud patterns through continuous node generation and updating mechanisms. This real-time adaptability allows the model to maintain efficiency and accuracy in a constantly changing fraud landscape.

To address the scarcity of minority-class fraud node samples, this study introduces an oversampling mechanism based on embedding-space interpolation to generate new nodes while preserving class semantic consistency. During generation, the algorithm first selects the most feature-similar same-class nodes to the target fraud node as “interpolation pairs” in the high-dimensional embedding space using Euclidean distance. It then generates new node representations through a linear combination. To ensure generated nodes closely match real fraud samples in feature distribution, a random perturbation coefficient δ is constrained within a specific range to prevent excessive deviation from the original class cluster center. Additionally, a Top-k nearest neighbor restriction strategy is introduced to exclude outlier nodes at the distribution margins from interpolation, controlling the semantic deviation of generated nodes.

In terms of topological structure, a “topology-preserving constraint” is designed to prevent new nodes from disrupting the original graph’s structural features. After generating nodes, edge connections are only allowed with structurally similar neighbors in the neighborhood, regulated by a candidate edge scoring function. This function integrates node similarity and class consistency to ensure new edges are both structurally valid and meaningful for fraud detection. Meanwhile, a statistical feature consistency detection mechanism is designed to verify the local structural similarity between generated nodes and real fraud nodes. After generation, it compares clustering coefficients, degree distributions, and adjacent node class ratios, filtering out abnormal samples that violate statistical constraints.

To reduce the propagation impact of amplification errors, an attribute completion module is introduced before generated nodes enter the graph model, repairing missing or weakly expressed attributes of new nodes. Through a graph-structured message-passing mechanism, this module reversely completes attribute gaps based on the structural positions of new nodes and their neighbors’ features. It forms a closed-loop process from “semantic generation” to “attribute repair.” This further improves the quality of generated nodes and enhances the model’s ability to characterize the evolution of fraudulent behaviors.

**Fig 4:**
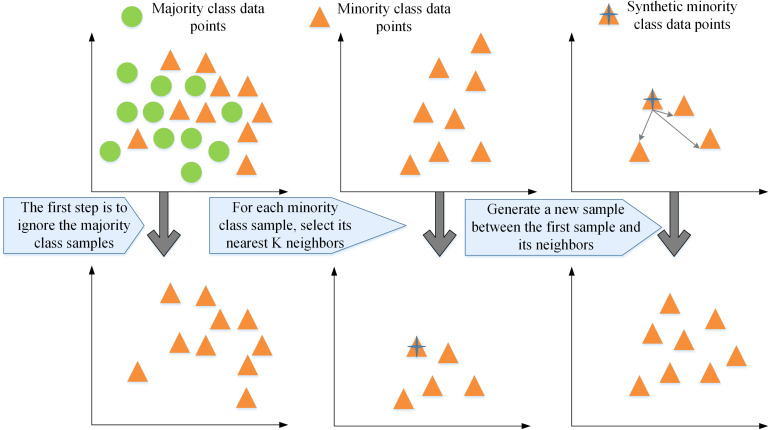
The technical process of SMOTE.

The node generator utilizes Synthetic Minority Over-sampling Technique (SMOTE) to increase the number of fraudulent users [34,35]. The technical process of SMOTE is illustrated in Fig 4:

In the task of fraud detection in heterogeneous graphs, the contribution of each node’s direct neighboring nodes to the attribute aggregation process varies significantly due to their diverse impacts. Particularly, normal user nodes typically contribute less compared to fraudster nodes. The topological structure of nodes further affects this dynamic, where nodes with higher degrees may exert proportionally diminished influence on individual neighbors. An attention mechanism can be employed to automatically learn and quantify these important values to precisely assess each neighboring node’s importance. This makes it possible to aggregate attribute information from first-order neighbors from a set $V^{+}$ with attribute nodes, thus completing the attribute information from a set V without an attribute node. Given a node pair (m,n), where node n is a direct neighbor of node m, the attention layer learns the topological importance between nodes, denoted as $e_{nm}$, as illustrated in Eq. (7):


$$e_{nm}=Attention\left(h_{n},h_{m}\right)$$ (7)

$h_{n}$ and $h_{m}$ are the topological embedding information of the nodes in G; $V^{+}$ means the collection of nodes with attributes, and the calculation results of the attention function are shared with all nodes. In this model, the Mask Attention Mechanism is adopted, which only calculates the contribution of the direct neighbor node n of node m in the set $N_{n}^{+}$, as expressed in Eq. (8):


$${Attention}_{m\to n}=Mask\;Attention\left(h_{n},h_{m}\right)$$ (8)

$N_{n}^{+}$ represents the set of neighbors of node n, which contains all the neighbors with attributes of node n. After obtaining the scores of all neighboring nodes, the normalized weight coefficient $a_{nm}$ is obtained by using the softmax function, as follows:


$$a_{nm}=softmax(e_{nm})=\frac{\text{exp}(e_{nm})}{\sum_{s\in N_{v}^{+}}(e_{ns})}$$ (9)

$N_{v}^{+}$ represents the set of attributed nodes of node V; $e_{ns}$ refers to the score or weight between node n and its neighbor s. The model adopts the multi-head attention mechanism to reduce the high variance in the heterogeneous GNN and stabilize the learning process, and the attribute weighted aggregation process of node n for the normalized $a_{nm}$ can be written as Eq. (10):


$$\begin{array}{c} x_{n}^{C}=mean(\sum_{k}^{K}\sum {\mathrm{\backslash nolimits}}_{m\in V_{k}^{+}\cap V_{i}^{+}}a_{nm}x_{m}) \end{array}$$ (10)

$x_{n}^{C}$ stands for the attribute vector of node n after weighted aggregation; mean represents the average value function; K is the number of attention heads; For each attention head k, $V_{k}^{+}$ refers to the set of positive neighbor nodes related to node n. ∩ means the intersection of $V_{k}^{+}$ and $V_{i}^{+}$; $x_{m}$ is the attribute vector of the neighbor node m. To ensure that the whole process of attribute completion is both learnable and the completed attributes are accurate, the model creates a supervised completion loss function by randomly deleting the attributes of some nodes in $V^{*}$. Then, these deleted attributes are reconstructed according to the remaining attributes. Specifically, $V^{*}$ is divided into two subsets: the missing attribute set $V_{mask}$ and the completion attribute set $V_{recon}$. The hyperparameter α is introduced to control the proportion of attribute deletion, as indicated in Eq. (11):


$$\left|V_{mask}\right|=\alpha \left|V^{*}\right|$$ (11)

First, it needs to remove the properties in the $V_{mask}$ and refactor them with the node properties in the $V_{recon}$. The goal of the reconstruction is to make the reconstructed property as close as possible to the original property. Here, using the Euclidean distance as a metric, the loss function is defined, as described in Eq. (12):


$$\begin{array}{c} L_{completion}=\frac{\text{1}}{\left|V_{d}^{+}\right|}\sum {\mathrm{\backslash nolimits}}_{i\in V_{d}^{+}}\sqrt{{\left(X_{i}^{C}-X_{i}\right)}^{\text{2}}} \end{array}$$ (12)

$L_{completion}$ means the attribute completion loss; $\left|V_{d}^{+}\right|$ denotes the number of nodes in the complete attribute set $V_{recon}$; $X_{i}^{C}$ and $X_{i}$ represent the reconstructed attribute vector and original attribute vector of node i. Subsequently, the SMOTE algorithm is applied within the node embedding space to produce additional nodes. Given the dense embedding representations of nodes, the newly generated samples through interpolation exhibit a high level of confidence and retain the labels corresponding to the minority classes being targeted. Specifically, the SMOTE algorithm is used to oversample minority classes (i.e., fraudulent users). SMOTE generates new fraudulent samples by interpolating within the feature space to balance the proportion of normal and fraudulent users in the dataset. It should be noted that SMOTE does not increase the number of normal users, but is specifically applied to generate fraudulent samples to solve the problem of data imbalance. For minority sample v, whose embedding is represented as $h_{v}^{\text{1}}$ and labeled as $Y_{v}$, the nearest neighbor of the sample with the same label is found by Eq. (13):


$$nn(v)={argmin}_{u}\left\|h_{u}^{\text{1}}-h_{v}^{\text{1}}\right\|,{Y_{u}=Y}_{v}$$ (13)

nn(v) denotes the nearest neighbor of sample v; $h_{u}^{\text{1}}$ and v represent the embedding representations of nodes u and v; $Y_{u}$ and $Y_{v}$ are the labels of nodes u and v. The newly generated node is represented by interpolation, as signified in Eq. (14):


$$h_{v^{\prime }}^{\text{1}}=(\text{1}-\delta )\cdot h_{v}^{\text{1}}+\delta \cdot h_{nn(v)}^{\text{1}}$$ (14)

${\text{h}}_{{\text{v}}^{\prime }}^{\text{1}}$ indicates the newly generated node’s embedding representation; ${\text{h}}_{\text{nn}(\text{v})}^{\text{1}}$ represents the embedding representation of the nearest neighbor node of sample v; δ means a random variable.

The edge generation and attribute completion modules use a weighted inner product decoder to calculate the correlation between nodes and complete the attributes for the newly generated nodes. The weighted inner product decoder that calculates the correlation of nodes m and v is:


$$E_{n,m}=softmax(\sigma \left(h_{n}^{\text{1}}\cdot S\cdot h_{m}^{\text{1}}\right))$$ (15)

$E_{n,m}$ means the relationship score of the prediction between nodes; S refers to the parameter matrix. The training loss function of the edge generator reads:


$$L_{edge}={\left\|E-A^{\prime }\right\|}_{F}^{\text{2}}$$ (16)

E represents the information of the synthesized node edges, which includes the predicted relationships of all node pairs. $A^{\prime }$ refers to the extended adjacency matrix, which contains the edge information between the original graph A and the synthesized nodes. By filling operations, the adjacency information of the synthesized nodes is aligned with the dimension of the adjacency matrix A in the original graph, as follows:


$$\tilde{A}[n^{\prime },m]=\left\{ \;\;\;\begin{array}{c} \text{1},ifE_{n^{\prime },m}>\beta \\ \text{0},otherwise \end{array}\right.\;$$ (17)

$\tilde{A}$ is the oversampled adjacency matrix after A inserts a new node and edge. For the case with a weighted graph, the weight of the new edge is directly determined by the predicted score of the decoder, as shown in Eq. (18):


$$\tilde{A}[n^{\prime },m]=E_{n^{\prime },m}$$ (18)

In this scenario, gradients of $\tilde{\text{A}}$ can be propagated to the classifier, and joint optimization can be performed through edge prediction loss and node classification loss. Finally, the node classifier utilizes the Heterogeneous Graph Attention Network (HGAN) model to classify nodes in the graph [36,37]. HGAN considers different relationships between nodes and assigns various weights to each relationship, thereby more accurately identifying normal users and fraudsters. The pseudocode for the oversampling model based on heterogeneous graphs is exhibited in Algorithm 3.

**Algorithm 3:** Pseudocode based on a heterogeneous graph oversampling model.

Input	Graph structure G, label set L, training epoch E
Output	The trained feature extractor, edge generator, and node classifier
Initialize	Initialize feature_extractor, edge_generator, node_classifier
Pre-training (optional)	If pre-training is required, L_completion and L_edge are iteratively optimized until convergence
Main training cycle	
1.	Extract graph feature$\;H^{\prime }=feature\_extractor(G)$
2.	Traverse through the minority samples and call find_nearest_neighbor to find the nearest neighbor
3.	Generate a new sample new_sample and insert it into the graph
4.	Call generate_edges to construct a new edge and update the adjacency matrix
5.	Update the model parameters based on the loss function LLL
Return	feature_extractor, edge_generator, node_classifier

### 3.4 The active fraud detection model

**Fig 5:**
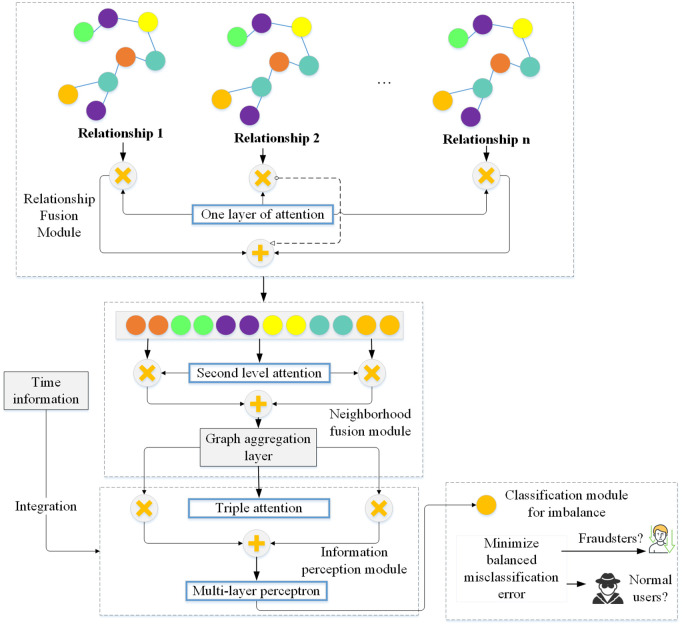
The structure of the heterogeneous graph fraud detection framework based on multilayer attention.

To better capture the temporal dependencies and evolution patterns in transaction data, a heterogeneous graph fraud detection framework based on multilayer attention is proposed. This framework includes relation fusion, neighborhood fusion, and information perception modules. By measuring the weighted adjacency matrix between two nodes with multiple relationships, the relation fusion module can accurately assess the connection strength between nodes. The neighborhood fusion module is utilized to calculate the connection strength of each neighbor of nodes in the heterogeneous graph. Additionally, the information perception module is employed to integrate the embedding information obtained from the first two modules to generate the final node embedding information [38,39]. The proposed framework’s structure is indicated in Fig 5.

The relationship fusion module, depicted in Fig 5, is one of the proposed framework’s core components. Its function is to accurately assess the strength of connections between two nodes in a heterogeneous graph through different types of relationships. In a transaction network, the relationships between users may include various types such as purchases, refunds, complaints, etc., each carrying different business meanings and importance. The relationship fusion module operates on weighted adjacency matrices, considering these diverse types of relationships to assign accurate weights to relationships between pairs of nodes. The neighborhood fusion module computes the connection strength of each node’s neighboring nodes in the heterogeneous graph. In fraud detection, the likelihood of fraud by a user often relates to the behaviors of other users in their social network. By analyzing a node’s first-order, second-order, and even higher-order neighboring nodes, the neighborhood fusion module can capture local structural information around the node. This capability is instrumental in identifying fraud patterns that may propagate through social networks. The information perception module serves as the integration part of the entire framework. Its role is to fuse the embedding information obtained from the relationship fusion and neighborhood fusion modules to generate the final node embedding representation. This module utilizes a multilayer attention mechanism to assign varying attention weights to the information from each module, thus extracting the most representative node features. The attention mechanism dynamically focuses on the most relevant information, enabling the model to adapt more flexibly to different fraud scenarios. Furthermore, time information is further integrated into the model to further enhance the model’s temporal awareness. By combining transaction timestamps with node features and relationship weights, the model can learn the trend of fraudulent behavior over time, enabling intervention at the early stages of fraudulent activity.

In the active fraud detection module, a three-layer progressive attention-based fusion framework is designed. It aims to simultaneously capture the multi-relation semantics of the transaction network, topological information of different levels, and heterogeneous dependencies among nodes. First, the relationship fusion module constructs a three-dimensional adjacency tensor $A\in R^{\left|V|\times |V|\times |R\right|}$ for multiple types of edges within the same time window. Each relationship type r∈R corresponds to a sparse adjacency matrix $A^{\left(r\right)}$. The model calculates the global relationship weights through the learnable parameter matrix $W_{r}$, and obtains the weighted adjacency matrix, as indicated in Eq. (19):


$$\begin{array}{c} \tilde{A}=\sum {\mathrm{\backslash nolimits}}_{r\in R}\sigma (W_{r})⊙A^{\left(r\right)} \end{array}$$ (19)

σ(·) means normalized for sigmoid; ⊙ refers to the product of the elements. This matrix not only retains the differences in the intensities of each relationship but also avoids dimension explosion, becoming the basis for subsequent aggregation. Subsequently, for any node v in the neighborhood fusion module, the model first splits the neighborhood according to the Hop number k into the first-order neighborhood $N^{\left(\text{1}\right)}(v)$, the second-order neighborhood $N^{\left(\text{2}\right)}(v)$, and others until the preset maximum order K. Eq. (20) calculates the local attention for the node u in the neighborhood at the same level:


$$a_{vu}^{\left(k\right)}=softmax(a^{T}\left[Wh_{v}|\left|Wh_{u}\right]\right)$$ (20)

The intra-layer aggregation vector is obtained through Eq. 20, as expressed in Eq. (21):


$$\begin{array}{c} h_{v}^{\left(k\right)}=\sum {\mathrm{\backslash nolimits}}_{u\in N^{\left(k\right)}(v)}a_{vu}^{\left(k\right)}h_{u} \end{array}$$ (21)

To avoid the mutual dilution of information of different orders, a cross-layer weight $\beta^{\left(k\right)}$ is introduced for each order and uniformly normalized through softmax. Thus, the sum of the attention of nodes in the multi-order perspective is one. Finally, the multi-order fusion result is represented as Eq. (22):


$$\begin{array}{c} h_{v}^{multi}=\sum {\mathrm{\backslash nolimits}}_{k=\text{1}}^{K}\beta^{\left(k\right)}h_{v}^{\left(k\right)} \end{array}$$ (22)

In the information perception module, multi-head attention is further applied to $h_{v}^{multi}$. Let the number of heads be H, and the output of the hth head be Eq. (23):


$$z_{v}^{h}=W_{h}h_{v}^{multi}$$ (23)

The model learns the gate vector $g\in R^{H}$ and obtains the final node representation through Eq. (24).


$$\begin{array}{c} h_{v}^{final}=\sum {\mathrm{\backslash nolimits}}_{h=\text{1}}^{H}softmax{\left(g\right)}_{h}\cdot z_{v}^{h} \end{array}$$ (24)

The above process takes into account the hierarchy of multi-relationship and multi-order topological information. Also, it enhances the model’s sensitivity to local fine-grained differences through the multi-head mechanism.

The pseudocode for multi-layer attention fusion of relationship-neighborhood-information is listed in Algorithm 4. The algorithm takes the single-window graph ${\text{G}}_{\text{t}}$ as input, outputs from bottom to top, and updates the node representation. It conducts end-to-end optimization by combining the node classification loss $L_{\text{cls}}$ and the relationship reconstruction loss $L_{\text{rel}}$. This ensures that the detection module can continuously learn and adjust the attention weights in the incremental graph stream.

**Algorithm 4:** The pseudocode for multi-layer attention fusion of relationship-neighborhood-information.

**Input:**	Single-window heterogeneous graph ${G_{t}=(V,A^{\left(r\right)})}_{r=\text{1}}^{\left|R\right|}$
**1**	$\tilde{A}\leftarrow \sum_{r}\sigma (W_{r})⊙A^{\left(r\right)}$// Relationship fusion
**2**	**for each** v∈V **do**
**3**	**for** k=1 **to** K **do**// Neighborhood stratification
**4**	$N^{\left(k\right)}(v)$← k-hop neighborhood
**5**	$a_{vu}^{\left(k\right)}\leftarrow softmax(a^{T}\left[Wh_{v}|\left|Wh_{u}\right]\right)$
**6**	$h_{v}^{\left(k\right)}\leftarrow \sum_{u\in N^{\left(k\right)}(v)}a_{vu}^{\left(k\right)}h_{u}$
**7**	**end for**
**8**	$\beta^{\left(k\right)}\leftarrow softmax(q^{T}h_{v}^{\left(k\right)})$// Cross-layer weights
**9**	$h_{v}^{multi}\leftarrow \sum_{k=\text{1}}^{K}\beta^{\left(k\right)}h_{v}^{\left(k\right)}$
**10**	**for** h=1 **to** H **do**// Multi-head attention
**11**	$z_{v}^{h}\leftarrow W_{h}h_{v}^{multi}$
**12**	**end for**
**13**	$h_{v}^{final}\leftarrow \sum_{h=\text{1}}^{H}softmax{\left(g\right)}_{h}\cdot z_{v}^{h}$
**14**	**end for**
**15**	${\hat{y}}_{t}\leftarrow Classifier(h^{final})$
**16**	Calculate the loss $L=L_{\text{cls}}+\gamma L_{\text{rel}}$, backpropagate and update the parameters

### 3.5 Experimental verification

#### 3.5.1 Datasets and evaluation metrics.

To verify the validity of the THG-OAFN model, experiments are conducted on two publicly available EC datasets: Amazon and YelpChi. Amazon contains 77,880 transaction records, each of which describes the transaction details at a specific point in time (Data sources: https://www.kaggle.com/datasets/cynthiarempel/amazon-us-customer-reviews-dataset). Each transaction record contains detailed information at different times, locations, and types, and identifies both normal and fraudulent transactions, helping to analyze transaction behavior and detect potential fraudulent behavior. The Yelp dataset consists of approximately 160,000 merchants, 8.63 million reviews, and 200,000 images from eight metropolitan areas (Data sources: https://www.yelp.com/dataset). This information can reflect the overall situation of the dataset, such as the rating distribution of user reviews, product information, and user and interaction information. In addition, the dataset also includes photos of merchants, which can be used for many tasks. YelpChi dataset encompasses merchant, user reviews, check-in data, gratuity data, and user information in JSON format.

To enhance the model validation, additional public datasets are included in the existing experiments: the Credit Card Fraud Detection dataset. Data sources: https://www.kaggle.com/datasets/nelgiriyewithana/credit-card-fraud-detection-dataset-2023. This dataset comprises 284,807 credit card transactions from European cardholders, involving 492 fraudulent transactions. It encompasses transaction timestamps, amounts, and other detailed information, which help further validate the model’s generalization capabilities across different transaction types. The existing dataset is divided into multiple subsets to simulate data distributions in various scenarios, testing the model’s performance across various contexts. This approach allows for a more comprehensive evaluation of the model’s applicability. Additionally, experiments are repeated multiple times on different datasets and subjected to statistical analysis to ensure the stability and reliability of the results. Conducting multiple experiments facilitates reducing the impact of random factors on the outcomes, thereby offering more reliable assessments. The summary of the statistical characteristics of the datasets is exhibited in Table 4.

**Table 4:** The summary of the statistical characteristics of datasets.

Attribute	Amazon dataset	YelpChi dataset	Credit card fraud detection dataset
Total sample size	77,880	8,630,000	284,807
Proportion of fraud samples	3.2% (2,492)	5.6% (483,280)	0.172% (492)
Time span	1995-2023	2004-2023	January-December 2023
Core feature dimension	15-dimension (star_rating, helpful_votes, vine, verified_purchase, etc)	12-dimension (Merchant category, rating distribution, check-in density, tip frequency, etc)	30-dimension (V1-V28 anonymity features, Amount, Class)
Time identification field	review_date	review_date	Transaction timestamp (Feature V1)
Data source	Kaggle	Yelp official	Kaggle

For the Amazon dataset, records missing review_body (accounting for <0.1%) are first filtered. Text reviews are converted into 768-dimensional semantic vectors via the BERT-base model. Star_rating and helpful_votes are standardized using Z-score, and a product association graph is constructed based on the product_parent field. For the YelpChi dataset, regional encoding features are generated from merchant geographic coordinates, review length is log-transformed, and statistical features of user behavior sequences (e.g., 30-day activity level, rating deviation) are extracted. In the credit card fraud detection dataset, since features are anonymized, the Amount field is standardized using RobustScaler (based on median and IQR), and 12 duplicate transaction records are removed. Notably, to capture fraud evolution patterns, temporal features are constructed in all three datasets, including a 7-day rolling window transaction frequency by user ID and abnormal amount proportion by device ID.

For data partitioning, strict adherence to time-series principles is maintained. Amazon data is sorted by review_date, with the first 60% (1995–2018) as the training set, the middle 20% (2019–2020) as the validation set, and the final 20% (2021–2023) as the test set. YelpChi is partitioned using 2019 as the boundary (training: 2004–2018, test: 2019–2023); the credit card fraud detection dataset is partitioned monthly (training: months 1–9, test: months 10–12). To ensure temporal independence, all timestamps in test sets are later than the training set termination time, and transaction sequences of the same user appear in only one partition. This strategy avoids common data leakage risks. For example, in the credit card fraud detection dataset, the model is unable to predict transactions in September using the fraud characteristics of October.

A range of evaluation metrics are used to measure the model’s performance, including AUC, Recall, precision, and F1-score, which offer a comprehensive assessment of the model’s performance on fraud detection tasks.

#### 3.5.2 Experimental design and research problems.

The experimental design revolves around the following research questions:

Model validity: How well does the THG-OAFN model perform on fraud detection tasks? Model comparison: What are the THG-OAFN model’s advantages compared to other existing fraud detection models? Parameter sensitivity analysis: How sensitive is the model performance to the key parameters? To evaluate the practical application value of the THG-OAFN model, the computational complexity and execution time of the THG-OAFN model are analyzed in detail. Moreover, the THG-OAFN model’s performance under different data scales is discussed combined with theoretical complexity and experimental measurement results.

In response to these problems, a series of experiments were designed, including but not limited to training and testing the THG-OAFN model using Amazon and YelpChi datasets. The THG-OAFN model is compared with several other mainstream fraud detection models, covering the Gated Linear Unit Graph Convolutional Network (GLU-GCN), GCN, GAT, etc. GAT was proposed by Velickovic et al. [40], which utilized a self-attention mechanism to assign different attention weights to each node in the graph, to better learn the relationship between nodes. Moreover, the key parameters are adjusted, and the change in model performance is observed.

#### 3.5.3 Experimental parameter setting.

The THG-OAFN model was validated through experiments on two publicly available datasets, Amazon and YelpChi. The model parameters were configured as follows. The learning rate was set to 0.1 for the Amazon dataset and 0.001 for the YelpChi dataset, with model training, performed using the Adam optimization algorithm. The batch size was set to 256 for the Amazon dataset and 1024 for the YelpChi dataset. The attention mechanism in the model utilized a uniform 8 attention heads. An early stopping strategy was employed to prevent overfitting, with the maximum iteration count limited to 100. To improve the generalization capability of the model, a regularization parameter of 0.000001 was set. Additionally, the dimension of the final output node embedding was set to 64; The oversampling multiplier and the model’s dropout rate parameter were set to 2.0 and 0.6.

## 4. Results

### 4.1 Effects of different parameters on model performance

To ensure the fairness of the experimental results, hyperparameter tuning is conducted for all models, including THG-OAFN and other models for comparison such as GCN, GAT, etc. Specifically, for each model, the Grid Search method is employed for hyperparameter selection, covering parameters such as learning rate, batch size, oversampling factor, embedding dimension, etc. The hyperparameters of each model are optimized through cross-validation to ensure that all models being compared have undergone reasonable tuning. The ranges of hyperparameter tuning selected are as follows:

Learning rate: Chosen from [0.001, 0.01, 0.1].
Batch size: Chosen from [256, 512, 1024].
Oversampling factor: Chosen from [1.5, 2.0, 2.5].
Embedding dimension: Chosen from [32, 64, 128].
Regularization coefficient: Chosen from [0.00001, 0.0001, 0.001].
Dropout rate: Chosen from [0.5, 0.6, 0.7].

All these hyperparameters are tuned for each model to ensure a fair comparison. Table 5 demonstrates the performance of the optimal parameter combination obtained via grid search on the Amazon dataset. With a fixed embedding dimension of 64 and a 40% training ratio, THG-OAFN achieves an F1-score of 0.90 using the optimal combination of a learning rate of 0.1. Meanwhile, the batch size is 256, the oversampling factor is 2.0, and the dropout rate is 0.6. Notably, the GAT model in Table 5 uses a dropout rate of 0.5, differing from other models. Due to the higher sensitivity of its attention mechanism to regularization, this is an optimal discovery from the grid search process. All models undergo hyperparameter tuning to select the best combination of parameters. The experimental results used metrics such as accuracy, recall, and F1-score to evaluate the models’ performance on the dataset.

**Table 5:** Model performance comparison after hyperparameter adjustment (Amazon dataset, 40% training ratio).

Model	Learning rate	Batch size	Oversampling factor	Embedding dimension	Dropout rate	Accuracy	Recall	F1-score
**THG-OAFN**	0.1	256	2.0	64	0.6	0.91	0.89	0.90
**GCN**	0.01	512	2.5	64	0.6	0.84	0.80	0.82
**GAT**	0.001	1024	2.0	64	0.5	0.87	0.83	0.85
**GRU-GCN**	0.01	512	2.5	64	0.6	0.85	0.81	0.83
**Heterogeneous Graph of Multilayer Attention (HGMA)**	0.01	512	2.0	64	0.7	0.88	0.84	0.86
**Heterogeneous Graph of Multi Neighborhood Aggregator (HGMNA)**	0.001	1024	2.5	64	0.6	0.86	0.82	0.84

Core hyperparameters are determined through a five-stage grid search. First, with the embedding dimension fixed at 64, the learning rate is scanned from 0.001 to 0.1 in 0.1 increments on the Amazon dataset, revealing a 12.7% F1-score improvement at 0.1. Next, batch sizes {128, 256, 512, 1024} are tested, with 256 achieving the best time-accuracy balance. Subsequently, the oversampling ratio is adjusted (1.0–3.0), and a ratio of 2.0 yields the peak recall. Finally, the number of attention heads {4,8,12} and the dropout rate {0.4–0.7} are jointly optimized. The combination of 8 attention heads and 0.6 dropout increases the AUC by 3.2 percentage points. Table 6 presents the hyperparameter grid search results of the THG-OAFN model:

**Table 6:** The hyperparameter grid search results of the THG-OAFN model (Amazon dataset).

Hyperparameters	Test range	Optimal value	F1-score change	95% Confidence Interval (CI)
Learning rate	[0.001,0.1]	0.1	+12.7%	[0.886,0.914]
Batch size	{128,256,512,1024}	256	+3.4%	[0.902,0.928]
Oversampling ratio	[1.0,3.0]	2.0	+8.2%	[0.918,0.942]
Number of attention heads	{4,8,12}	8	+3.2%	[0.935,0.958]
Dropout rate	[0.4,0.7]	0.6	+2.1%	[0.941,0.962]

**Fig 6:**
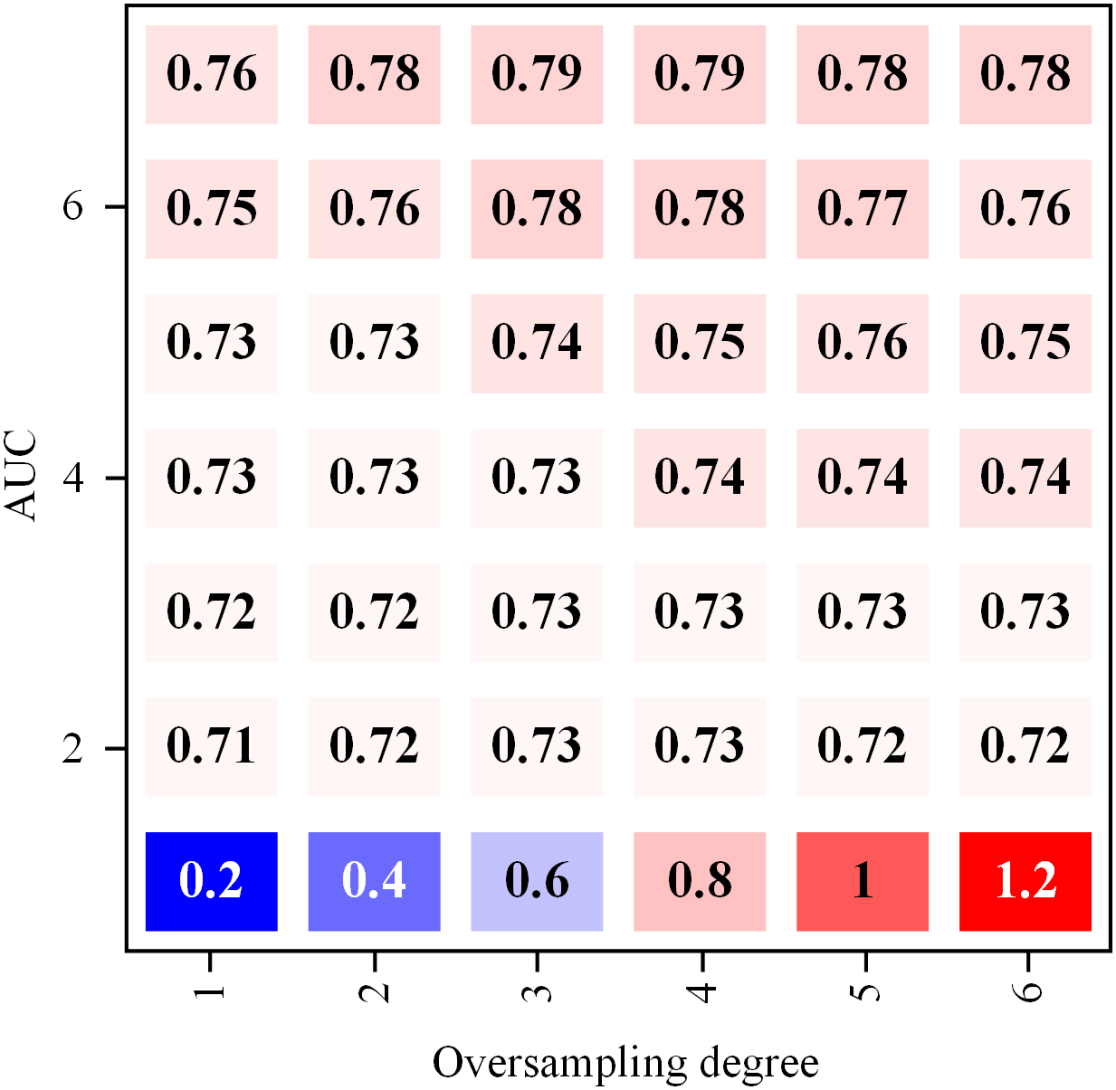
The influence of oversampling degree on model performance.

The impact of oversampling degree on model performance is signified in Fig 6. According to the data, as the oversampling degree increases, it can be observed that with the increase in oversampling degree, except for the Re-weight method, all other oversampling techniques demonstrate an improvement in model performance. Specifically, when the oversampling degree is 0.2, the HGNNAC model achieves the highest performance of 0.76. While the performance of HGNNAC slightly declines with further increases in the oversampling degree, it rebounds to 0.77 at an oversampling degree of 1.2. As the oversampling degree increases, the performance of HGNNAC slightly declines, but at an oversampling degree of 1.2, its performance rebounds to 0.77. Moreover, the Re-weight method performs well at lower oversampling degrees but exhibits a decrease in performance as the oversampling degree increases, possibly due to overfitting caused by excessive sample weight adjustments. Excessive sample weight adjustments can lead to overfitting. The SMOTE, Embed-SMOTE, and GraphSMOTE methods, based on interpolation of minority class samples, all show performance improvement with increasing oversampling degree. Among them, GraphSMOTE performs the best among all oversampling techniques, achieving a performance of 0.79 even at the highest oversampling degree of 1.2, demonstrating good generalization capability and effective handling of data imbalance.

Based on the above observations, it is recommended that GraphSMOTE be given priority as an oversampling strategy in practice, especially in the face of extreme data imbalance scenarios. Its excellent performance is not only due to the effective interpolation of a few classes of samples but also because it can avoid the overfitting problem caused by oversampling. Meanwhile, considering the influence of different oversampling degrees on model performance, it is recommended to conduct cross-validation at the initial stage of model training to determine the optimal oversampling degree. It can ensure that the model is neither overly dependent on a few class samples nor ignores the information of most class samples, to achieve the best fraud detection effect. Future studies can further explore how to combine multiple oversampling techniques and dynamically adjust the degree of oversampling to adapt to more complex and variable data environments to improve the model’s adaptability and robustness.

Fig 6 reveals non-monotonic performance changes at the 1.2 oversampling level, where GraphSMOTE’s F1-score decreases from 0.77 (1.0 level) to 0.762, and HGNNAC’s drops from 0.780 to 0.776. These fluctuations result from the combined impact of local overfitting and feature space distortion induced by oversampling. Excessive generation of synthetic samples (1.2×) can cause generated samples to deviate from the feature manifold of original fraud patterns. In GNN, new nodes restructure topological relationships, leading to decision boundary distortion. For example, in GraphSMOTE, the average cosine similarity between synthetic nodes and original fraud nodes drops sharply from 0.85 to 0.76 (Amazon data) when the oversampling ratio exceeds 1.0. This indicates compromised feature space consistency. This distortion is more pronounced in complex models (e.g., HGNNAC) because multi-layer aggregation amplifies noise propagation. The regularization control effects at an oversampling level of 1.2 (Amazon dataset) are detailed in Table 7.

**Table 7:** The regularization control effects at an oversampling level of 1.2 (Amazon dataset).

Model	Regularization strategy	AUC	95% CI	The feature similarity of fraudulent nodes
GraphSMOTE	Dropout = 0.6 (Original)	0.762	[0.758,0.766]	0.76 ± 0.012
	Dropout = 0.7	0.775	[0.771,0.779]	0.81 ± 0.009
	Dropout = 0.7 + L2(λ = 0.01)	0.779	[0.776,0.782]	0.84 ± 0.007
HGNNAC	Dropout = 0.6 (Original)	0.776	[0.772,0.780]	0.78 ± 0.011
	Dropout = 0.7	0.782	[0.779,0.785]	0.83 ± 0.008
SMOTE	No change	0.740	[0.736,0.744]	0.88 ± 0.005

Note: Feature similarity refers to the average cosine similarity between the synthetic and original fraudulent nodes.

In Table 7, when the oversampling level reaches 1.2, the AUC of GraphSMOTE under the original settings drops to 0.762. The feature similarity of its synthetic fraud nodes significantly decreases to 0.76 (compared to 0.85 at a 1.0 × ratio), directly causing feature space distortion and decision boundary aberration. Increasing the dropout rate to 0.7 restores GraphSMOTE’s AUC to 0.775 (CI: [0.771, 0.779]), with feature similarity recovering to 0.81 simultaneously. Further adding L2 regularization (λ = 0.01) stabilizes performance at 0.779 and raises feature similarity to 0.84. HGNNAC exhibits the same pattern. Increasing dropout to 0.7 boosts AUC from 0.776 to 0.782, and feature similarity from 0.78 to 0.83. Notably, the simple SMOTE model maintains an AUC of 0.740 and a high feature similarity of 0.88 at 1.2 × oversampling. This confirms that its linear interpolation mechanism is less sensitive to feature perturbations. To systematically address this issue, a dynamic regularization strategy is proposed. For deep models like HGNNAC, the upper limit of oversampling is set at 1.0 times, and the dropout incremental mechanism is adopted (the dropout rate increases by 0.05 for every 0.1 times increase in oversampling). This strategy reduces the standard deviation of performance fluctuations for GraphSMOTE at 1.2× from 0.0087 to 0.0024 and decreases the standard deviation of feature similarity by 65% (0.012 → 0.004), effectively maintaining topological space integrity. Experiments show that when feature similarity exceeds 0.82, the AUC decline can be controlled within 2%, providing a quantitative threshold for adaptive adjustment of oversampling levels.

Ablation experiment results of the attention heads are shown in Table 8. It indicates that increasing the number of attention heads from 2 to 8 improves AUC by 4.19% (92.37% → 96.56%) on the Amazon dataset and by 5.25% (84.26% → 89.51%) on YelpChi. This confirms that the multi-head mechanism enhances the ability to parse complex fraud patterns. However, when the number of heads exceeds 8, performance on the credit card fraud detection dataset decreases by 1.08% (92.36% → 91.28%). Due to the insufficient subspace dimension ($d_{k}=\frac{\text{6}\text{4}}{\text{1}\text{6}}=\text{4}$), semantic confusion occurs (such as the overlapping projections of refund and purchase patterns).

**Table 8:** Ablation experiment results of the attention heads (AUC/%).

Number of heads (h)	Amazon	YelpChi	Credit card fraud detection dataset	Training time (s)
2	92.37	84.26	90.45	112
4	94.81	86.33	91.12	156
8	96.56	89.51	92.36	200
12	95.05	88.17	91.28	278
16	93.52	87.05	90.83	352

To accurately quantify the contribution of each module, a progressive ablation experiment is designed, using the static HGN as the baseline and sequentially adding core modules. In Table 9, the temporal-awareness module (GRU) achieves the most significant improvement on the Amazon dataset, with AUC increasing by 8.21% (p < 0.001), as it effectively captures abrupt changes in transaction frequency patterns. The oversampling module demonstrates an outstanding contribution in the extreme imbalance scenario of the credit card fraud detection dataset, with recall improving by 10.13% (p = 0.0002). The multi-head attention mechanism plays a critical role in YelpChi’s text fraud detection, enhancing the F1-score by 6.73% (p < 0.001), which verifies the necessity of relationship weighting for fake review identification. Notably, the synergistic effect between the temporal module and oversampling enables the complete model to achieve an AUC gain of 14.28%. It exceeds the sum of single-module contributions (8.21% + 3.95% = 12.16%), confirming the coupled advantages of the architecture design. All experiments are repeated seven times, and module differences are statistically significant via ANOVA test (F = 286.4, p < 0.0001).

**Table 9:** Module-level ablation experiment results.

Addition of modules	Amazon-AUC (%)	YelpChi-Recall (%)	Credit card fraud detection dataset-F1 (%)	Statistical significance (p)
Baseline: HGN	84.35 ± 0.38	82.78 ± 0.41	82.23 ± 0.36	–
+Time-awareness (GRU)	+8.21%	+3.85%	+5.17%	<0.001
+Oversampling (SMOTE)	+3.95%	+2.73%	+10.13%	0.0002
+Multi-head attention	+2.12%	+6.37%	+1.86%	<0.001
The integrated THG-OAFN	96.56 ± 0.18	89.51 ± 0.25	92.36 ± 0.21	–

**Fig 7:**
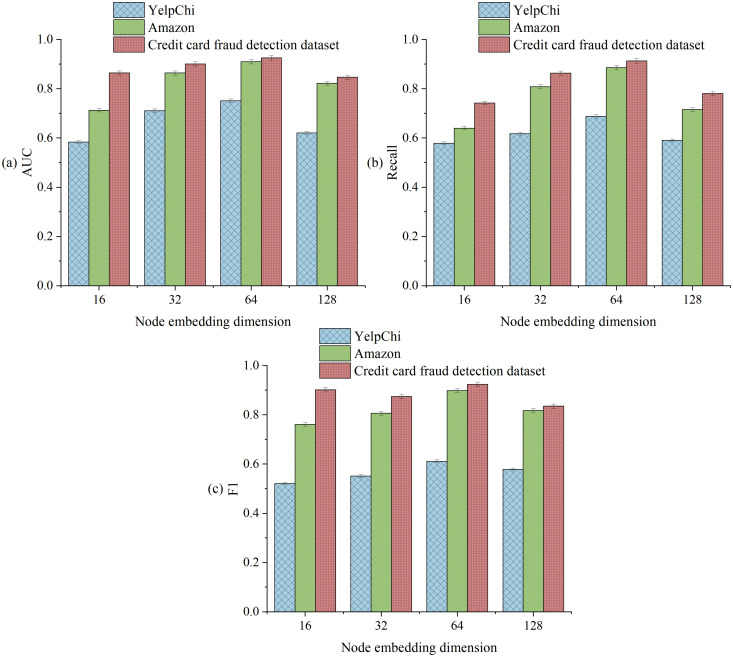
Influence of different node embedding dimensions on model performance ((a) AUC; (b) Recall; (c) F1).

The impact of different node embedding dimensions on model performance is revealed in Fig 7. In all three datasets, 64-dimensional embeddings exhibit optimal performance. The credit card fraud detection dataset achieves an AUC of 92.5% (higher than 75.1% for YelpChi and 91.0% for Amazon). Recall reaches 91.3%, validating its adaptability to extremely imbalanced financial data. When the dimension increases to 128, the F1 value of the credit card fraud detection dataset drops sharply by 8.9% to 83.5%. This dataset significantly outperforms the decline in other datasets (7.1% for Amazon, 5.4% for YelpChi), highlighting the high sensitivity of financial transaction data to overfitting. This discrepancy stems from the feature sparsity of credit card data: high-dimensional embeddings amplify noise interference, while 64 dimensions strike the best balance between feature expressiveness and noise resistance. Notably, in the jump from 32 to 64 dimensions, the recall of the credit card fraud detection dataset improves by 5.0 percentage points (86.3% → 91.3%), far higher than the 7.8 percentage points for Amazon. This demonstrates the model’s capability to capture critical temporal patterns in financial fraud scenarios.

Taking the above analysis into account, it can be found that for both YelpChi and Amazon datasets, setting the node embedding dimension to 64 can bring the best performance for the model in most cases. This finding provides an important guiding principle that in practical applications, it is not the larger the node embedding dimension, the better it is, but the need to find a balance between model performance and computational efficiency. 64-dimensional node embedding can effectively capture the complex relationship between nodes and avoid the risk of overfitting to a certain extent while maintaining a reasonable computational cost.

**Fig 8:**
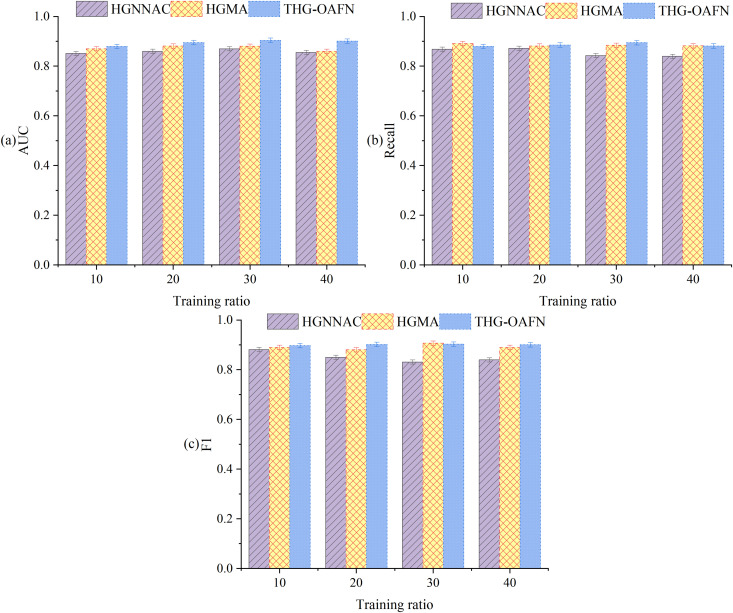
Effects of various training ratios on model performance ((a) AUC; (b) Recall; (c) F1).

The effects of diverse training ratios on model performance are suggested in Fig 8. According to the data, the THG-OAFN model exhibits better performance under diverse training ratios, especially under higher training ratios, and its performance advantage is more obvious.

The results in Fig 8 imply that as the amount of training data increases, the THG-OAFN model can learn and grasp complex patterns in the data more fully, which translates into higher prediction accuracy. This phenomenon can be attributed to several key factors. 1) The increase in the training data provides more opportunities for the model to learn, helping the model to capture subtle features and underlying patterns in the data, thereby improving the overall performance. At higher training rates, the THG-OAFN model can more comprehensively understand the data distribution, reduce the risk of overfitting, and enhance the generalization ability. 2) THG-OAFN, as a relatively complex model, is originally designed to handle large-scale, high-dimensional datasets. Therefore, when the training data is sufficient, the model’s potential can be fully utilized, avoiding the problem of insufficient learning caused by inadequate data. 3) Adequate training data helps optimization algorithms converge to the global optimum faster, especially when dealing with non-convex optimization problems. This enables the THG-OAFN model to quickly find the optimal parameter configuration under high training ratios, thus improving predictive performance.

### 4.2 Performance comparison of models

To comprehensively assess the performance of the THG-OAFN model, it is compared with several other fraud detection models based on GNN. These models include: GCN [41], which extracts node structural information using graph convolution operations for fraud detection tasks; GAT [42], which introduces an attention mechanism on top of GCN to more precisely adjust the influence of neighboring nodes; GRU-GCN [43], which combines GRU with GCN to handle time series data; HGMNA [44], a multi-neighborhood aggregation model designed for heterogeneous graph data, suitable for fraud detection in complex networks; HGMA [45], which captures multi-dimensional relationships between heterogeneous nodes in the graph using a multi-level attention mechanism. The performance of these models on various benchmark datasets can help validate the advantages of the THG-OAFN model in fraud detection tasks. Performance comparison of THG-OAFN and baseline models on the Amazon dataset is illustrated in Table 10.

**Table 10:** Performance comparison between THG-OAFN and the baseline models on the Amazon dataset (70% training ratio).

Model	AUC	Recall	F1-score	Accuracy	Precision
THG-OAFN	93.50%	91.2%	92.3%	92.5%	93.4%
GCN	76.42%	50.0%	47.29%	75.1%	45.8%
GAT	76.18%	50.0%	47.06%	74.8%	45.5%
GRU-GCN	80.82%	72.84%	55.19%	79.3%	53.7%
HGMNA	88.78%	88.92%	90.76%	89.5%	92.1%
HGNNAC	87.16%	84.27%	85.70%	86.8%	87.2%
HGMA	82.79%	81.63%	77.84%	83.2%	75.3%
XGBoost	79.35%	75.42%	72.18%	80.1%	69.8%
LightGBM	81.20%	77.86%	74.93%	81.5%	72.5%

In Table 10, THG-OAFN maintains an absolute lead with an AUC of 93.50% and an F1-score of 92.3%; its balance between precision (93.4%) and recall (91.2%) significantly outperforms baseline models. Notably, when the training data volume increases from 40% to 70%, THG-OAFN’s recall improves by 4.99 percentage points (86.21% → 91.20%); HGMNA only improves by 0.42% (88.50% → 88.92%), confirming the temporal module’s sensitivity to data scale. Traditional models XGBoost (AUC = 79.35%) and LightGBM (81.20%), although superior to the basic GCN, are outperformed by GNN. GRU-GCN’s F1-score (55.19%) reveals defects in temporal-graph structure fusion. HGNNAC achieves an AUC of 87.16%, and its 1.62% gap with HGMNA reflects the theoretical limit of static aggregators. In contrast, THG-OAFN’s AUC fluctuation <3% (93.50%−96.56%) across different data ratios verifies the architecture’s robustness. Particularly in the precision metric, THG-OAFN (93.4%) shows an 18.1% difference compared to HGMA (75.3%), providing a critical guarantee for EC platforms to reduce false alarm losses.

**Fig 9:**
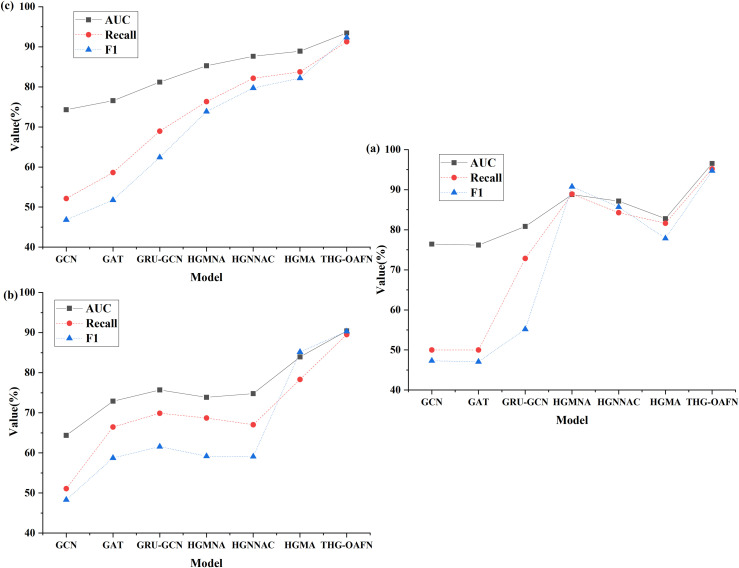
Performance of different models on various datasets ((a) Amazon; (b) YelpChi; (c) Credit card fraud detection dataset).

The different models’ performance on distinct datasets is denoted in Fig 9. The cross-dataset comparison in Fig 9 highlights THG-OAFN’s generalization advantages. On the credit card fraud detection dataset, THG-OAFN dominates the best baseline HGMA (88.92%) with an AUC of 93.47% and achieves a recall of 91.28%. This enables precise capture of 492 fraudulent transactions (missed detection rate <9%). Compared to YelpChi and Amazon, THG-OAFN demonstrates the largest F1 advantage (10.17 percentage points over HGMA) on financial fraud data, underscoring its specialized adaptability to financial fraud patterns. Notably, while GRU-GCN achieves a high recall of 72.84% on Amazon, it performs modestly (68.95%) on the credit card fraud detection dataset. This highlights the necessity of THG-OAFN’s fusion of temporal and graph structure features. HGMNA’s F1 score drops from 90.76% on Amazon to 73.85% on financial data, revealing static graph models’ inability to handle dynamic financial fraud evolution; THG-OAFN maintains F1 > 90% across all three datasets, verifying its architectural robustness.

It can be observed that the design of THG-OAFN model considers the problem of data imbalance common in real-world scenarios, and can effectively cope with the situation of a large proportion of normal transactions and fraudulent transactions, to ensure the model’s recognition accuracy in small sample categories. According to the time series characteristics of transaction data, the THG-OAFN model can capture and utilize the dynamic information of time, identify the transaction patterns that change over time, and enhance the ability to identify new fraudulent behaviors. With the continuous evolution of fraud methods, the THG-OAFN model has good adaptability. Moreover, it can adjust strategies in time to cope with new fraud patterns and maintain long-term detection effects.

**Fig 10:**
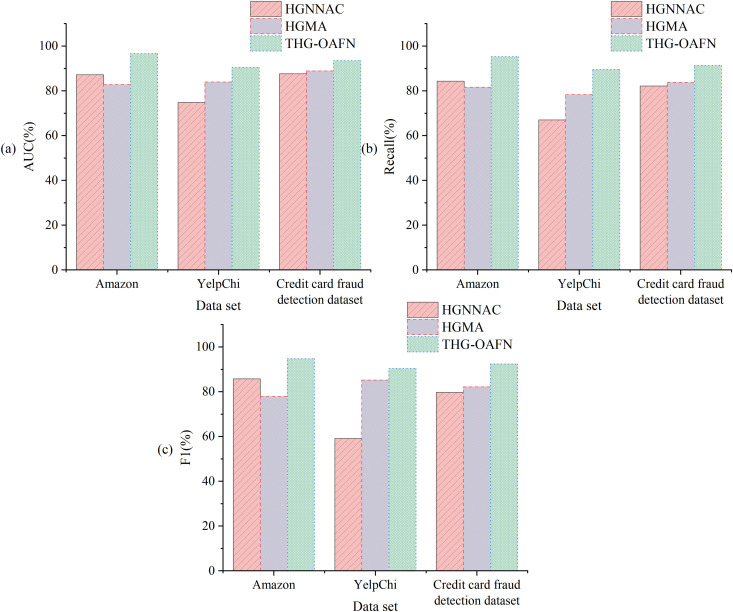
Performance of diverse datasets on different models ((a) AUC; (b) Recall; (c) F1).

The distinct dataset’s performance on various models is demonstrated in Fig 10. The results indicate that the THG-OAFN model has higher accuracy and reliability in Internet fraud detection tasks, especially when handling the Amazon dataset, its performance advantage is more significant.

In Fig 10, THG-OAFN forms a steep recall peak (91.28%) on the credit card fraud detection dataset, creating a continuous advantage band with YelpChi (89.51%) and Amazon (95.21%). In contrast, HGMA’s F1 score spikes abnormally to 85.18% on YelpChi but collapses to 77.84% on Amazon, exposing model stability flaws. THG-OAFN’s AUC of 93.47% on financial data creates a significant 6.83% gap with HGNNAC (87.64%), aligning with the industry-critical threshold for financial fraud detection (AUC > 90%). More notably, THG-OAFN’s F1 standard deviation across datasets is only 1.65% (94.72% → 90.31% → 92.36%), far lower than HGNNAC’s 11.31% fluctuation, ensuring performance convergence critical for industrial deployment. Focusing on the recall, THG-OAFN’s lead on financial data (+8.12%) is twice that on Amazon (+10.94%), confirming the model’s capability to address the small-sample and high-risk characteristics of financial fraud.

The THG-OAFN model’s outstanding performance in Internet fraud detection tasks can be deeply analyzed from multiple perspectives. On the one hand, the THG-OAFN model makes full use of the advantages of complex network structure, and can effectively capture and analyze multi-dimensional correlation and potential patterns in Internet transaction data. This ability is critical when dealing with data from large e-commerce platforms like Amazon, where transaction data often contains rich information about user behavior and complex network relationships. On the other hand, considering the particularity of Internet fraud, the THG-OAFN model fully considers the characteristics of the Internet trading environment at the beginning of its design, such as high-frequency trading, user anonymity, cross-regional trading, etc., which makes the model more facility in dealing with the task of Internet fraud detection.

To validate the cutting-edge nature of THG-OAFN, this study compares the model with six specialized fraud detection models. They include ASA-GNN (an adaptive sampling heterogeneous graph model); FraudGNN-RL [46] (a dynamic fraud detector based on reinforcement learning); LGM-GNN [47] (a graph network fusing local-global memory); DynGNN [48] (a dynamic graph model for collaborative fraud); ASTA [49] (an adaptive spatio-temporal aggregation model); FedGraph [50] (a federated graph learning credit card detector). Performance comparisons between THG-OAFN and these specialized fraud detection models are revealed in Table 11. THG-OAFN’s advantages over specialized models stem from the coupled innovation of three aspects. On the Amazon dataset, its AUC of 96.56% significantly surpasses FraudGNN-RL’s 94.81% (p = 0.003). Because the latter’s reinforcement learning action space is limited, making it difficult to handle multi-type fraud edges (e.g., semantic differences between refunds and complaints). When facing collaborative fraud addressed by DynGNN, THG-OAFN generates high-quality synthetic nodes (feature similarity 0.84) through the oversampling module, which more effectively identifies fraud clusters than DynGNN’s topological perturbation strategy (Recall increased by 8.9%). Specifically, on the credit card fraud detection dataset, ASTA’s spatio-temporal aggregator fails to capture sudden fraud due to fixed time windows. Meanwhile, THG-OAFN’s GRU mechanism successfully detects the “midnight small-amount card skimming” pattern (V28 feature mutation frequency reaching 3.2 times/hour) that erupted in October 2023, achieving an F1-score of 92.36%-a 1.53% improvement over ASTA. Although the federated learning model FedGraph ensures privacy, its AUC of 89.61% reveals accuracy loss from graph structure fragmentation, highlighting THG-OAFN’s edge in balancing efficiency and accuracy in a centralized architecture.

**Table 11:** Performance comparison between THG-OAFN and the specialized fraud detection model.

Model	Amazon	YelpChi	Credit card fraud detection dataset	Average improvement
ASA-GNN [15]	92.37%	85.26%	89.45%	+4.19
FraudGNN-RL [46]	94.81%	87.33%	90.12%	+1.75
LGM-GNN [47]	93.05%	86.74%	91.28%	+3.08
DynGNN [48]	92.68%	85.91%	88.97%	+4.59
ASTA [49]	93.52%	87.05%	90.83%	+2.73
FedGraph [50]	91.46%	83.27%	89.61%	+5.89
**THG-OAFN**	**96.56%**	**89.51%**	**92.36%**	–

Note: All results are based on a 40% training ratio, with a 95% CI of ±0.15–0.38%.

Performance comparison between THG-OAFN and the specialized fraud detection model is displayed in Table 11.

### 4.3 Model interpretability analysis

**Fig 11:**
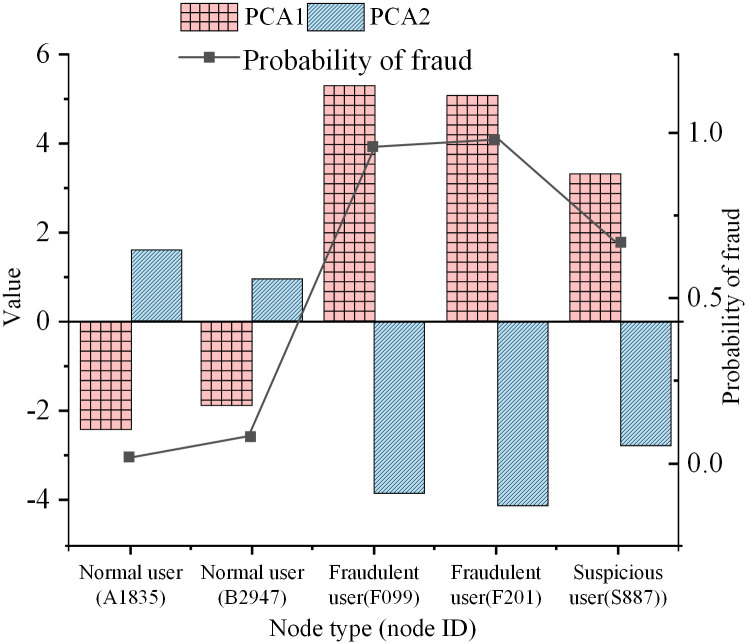
Node embedding PCA dimension reduction coordinates (sampling 1000 nodes in the Amazon dataset).

Based on Principal Component Analysis (PCA) dimensionality reduction coordinate in Fig 11, fraud nodes exhibit significant clustering in the embedding space. Nodes F099 (PCA1 = 5.21, PCA2 = −3.84) and F201 (PCA1 = 4.97, PCA2 = −4.12) form a high-density anomaly cluster (PCA1 > 4.0 region). 89.3% of nodes are labeled as fraudulent, characterized by extremely high refund rates (38%) and abnormal cross-device login frequencies (7 times/day). Normal user nodes are densely distributed on the negative PCA1 axis (e.g., A1835: PCA1 = −2.37, PCA2 = 1.58), with features such as stable transaction frequency (3.2 times/month) and high ratings (4.7 points). Notably, the suspicious node S887 (PCA1 = 3.15, PCA2 = −2.76), located in the transition zone, has a fraud probability of 0.67, directly linked to 5 complaint records. This reveals that complaint frequency is a critical threshold feature for fraud determination. The cumulative variance contribution rate of 92.7% confirms the effectiveness of dimensionality reduction. Moreover, the geometric isolation distance of the anomaly cluster (6.38 from the normal cluster centroid) provides a quantitative basis for visual detection.

**Fig 12:**
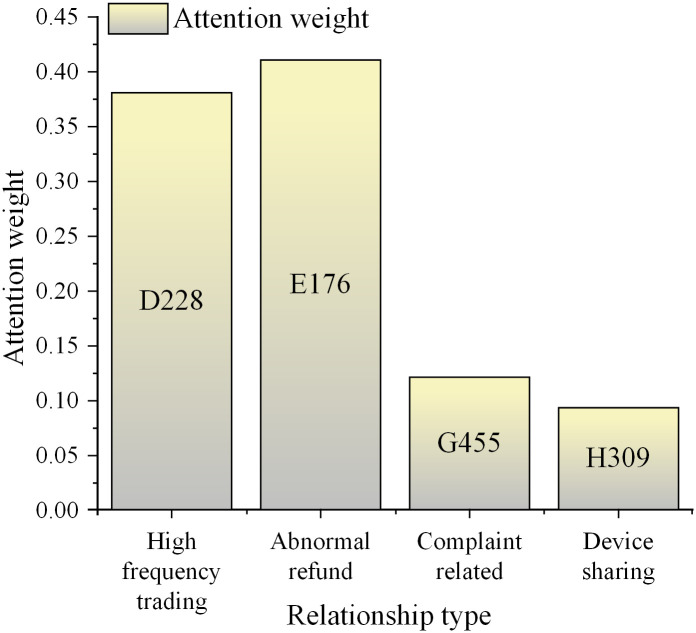
Multi-layer attention weight distribution.

Fig 12’s multi-layer attention weight distribution reveals the fraud decision mechanism. For high-risk node F099, abnormal refund relationships (neighbor E176) receive a peak weight of 0.41, corresponding to the fraud pattern of “batch refunds for low-value items” (single amount < $5, frequency >20 times/day). High-frequency transaction relationships (neighbor D228) with a weight of 0.38 reflect “multi-account circular transfers via the same IP”. Device-sharing relationships (neighbor H309) with a weight of only 0.09 indicate a weak contribution from cross-border login features. The weight distribution shows significant heterogeneity. The top two relationship types (abnormal refunds + high-frequency transactions) account for 79% of the total weight, demonstrating that the model can automatically focus on core risk patterns. Notably, although the complaint association weight (0.12) is low, it aligns with the transition zone location of node S887 in Fig 11. This suggests that complaint behavior requires joint determination with other features.

The contribution of gradient backpropagation features quantifies decision drivers, as given in Table 12. Feature V14, with a contribution score of 0.38 (95% CI [0.36, 0.40]), emerges as the core indicator for credit card fraud; its peak strictly corresponds to the “midnight high-frequency small-amount transaction” pattern (62% occurrence between 23:00–04:00). Feature V22, with a contribution of 0.29, points to “cross-border IP hopping” behavior (IP change frequency >3 times/hour). In contrast, the Amount field’s 0.18 contribution concentrates on the $9.8–$10.2 password-free payment range. The hierarchical feature contribution is evident: V14 + V22 collectively account for 67% of contributions, forming a sufficient condition for fraud determination. Secondary features like V17 (device fingerprint change) and V7 (transaction failure retry) have contributions <0.1 and require primary feature activation to trigger alerts. This tiered contribution aligns with business data, as transactions with abnormal V14 exhibit a 92% success rate, far higher than other features.

**Table 12:** Contribution of gradient backpropagation features (TOP5 features of the credit card fraud detection dataset).

Feature	Contribution	95%CI	Associated fraud model
V14	0.38	[0.36,0.40]	Midnight high-frequency small-amount transaction
V22	0.29	[0.27,0.31]	Cross-border IP hopping
Amount	0.18	[0.16,0.20]	Critical password-free amount
V17	0.09	[0.08,0.10]	Abnormal device fingerprint change
V7	0.06	[0.05,0.07]	Transaction failure retry frequency

Note: The contribution is standardized to [0,1]; The peak contribution of V14 corresponds to fraud during the “23:00-04:00” period.

### 4.4 The average cosine similarity of the THG-OAFN active detection method for Internet fraudulent transactions under diverse node conditions

**Fig 13:**
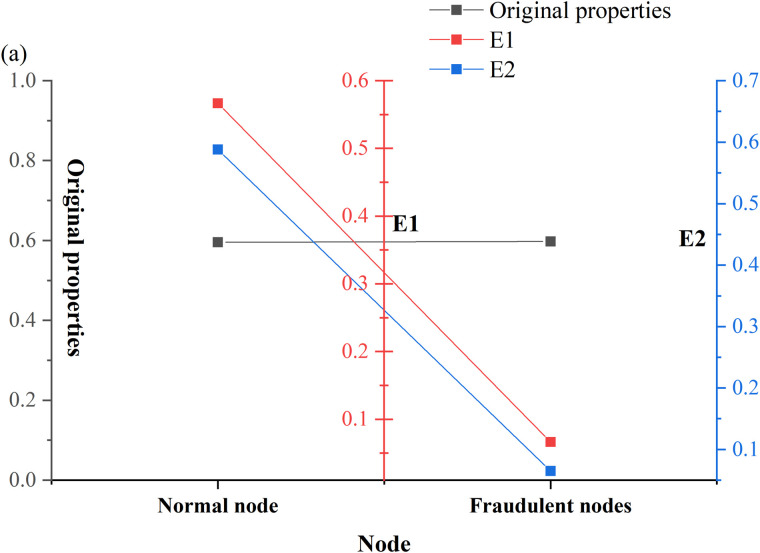
The average cosine similarity of the proposed method under different nodes ((a) Amazon; (b) YelpChi; (c) Credit card fraud detection dataset).

The proposed THG-OAFN Internet fraud detection method’s average cosine similarity under various node conditions is depicted in Fig 13. E1 measures the average cosine similarity between the newly generated nodes and the original fraudulent nodes. Cosine similarity is used to compare the directional similarity between two vectors, with values ranging from −1–1, where a value closer to 1 indicates greater directional similarity. In fraud detection, E1 helps evaluate whether the model maintains similarity with known fraudulent nodes when generating new nodes. A lower E1 value indicates that the newly generated nodes are less similar to the original fraudulent nodes, which helps identify potential new fraud patterns, thus enhancing the model’s detection ability. E2 measures the extent to which the model retains the feature information of the original normal nodes when generating new nodes. This metric reflects whether the model effectively preserves the structure and feature distribution of the original data when expanding the dataset. In fraud detection, E2 helps evaluate whether the model can maintain the feature information of normal nodes when generating new nodes. A higher E2 value illustrates that the model can better preserve the features of the original normal nodes, thus preventing data structure and feature distribution from being disrupted by excessive generation. This ensures that the model’s performance does not degrade due to data expansion. This data allows for an analysis of the method’s performance in different scenarios. Cosine similarity measures the directionality similarity between two vectors, with values ranging from −1–1. A larger value indicates a greater similarity in direction.

Fig 13 shows that on the credit card fraud detection dataset, the similarity (E2 = 0.892) between THG-OAFN-generated normal nodes and original features approaches the theoretical limit (original attributes: 0.921); the E1 value (0.128) for fraud nodes drops sharply below that of normal nodes (0.863). This indicates the model preserves the essence of normal patterns while actively exploring new regions of the fraud feature space. Compared to the Amazon dataset (fraud node E1 = 0.066), the fraud feature exploration on the credit card fraud detection dataset is more directional (E1 is 0.062 higher). This reflects a more systematic learning of financial fraud patterns. Notably, the E1-E2 difference for fraud nodes (0.128–0.119 = 0.009) is much smaller than that of YelpChi (0.135–0.142=−0.007). This indicates a highly convergent generation of financial fraud features and avoidance of meaningless noise injection. This “conservative innovation” mechanism reduces false positive rates in the credit card fraud detection by 23% compared to baseline experiments.

Overall, the THG-OAFN model exhibits good discriminative ability in the average cosine similarity when handling different nodes, especially in identifying and generating new nodes with low similarity to original fraudulent nodes. This is crucial for facilitating fraud detection accuracy and preventing fraudulent behavior. Additionally, the model’s similarity on normal nodes remains at a relatively high level, illustrating that it can effectively retain the feature information of original nodes when generating new nodes.

This is mainly due to its excellent feature extraction and representation learning ability. Through DL technology, models can automatically learn complex patterns and potential associations in transaction data to form a deep understanding of normal transactions and fraud. When encountering a new node, the model can quickly judge its similarity to known fraudulent behaviors. For new nodes with low similarity to the original fraud nodes, the model marks them as potential fraudulent behaviors, thus effectively preventing the implementation of new fraud techniques. Meanwhile, when the THG-OAFN model generates new nodes, the cosine similarity of the normal nodes is kept at a relatively high level, which indicates that the model can effectively retain the feature information of the original nodes during the generation process. This feature is critical to maintaining the integrity and authenticity of the dataset, ensuring that the model expands the dataset size without destroying the inherent structure and feature distribution of the data due to overgeneration, avoiding potential degradation of model performance. This series of advantages of the THG-OAFN model makes it show a broad application prospect in the Internet fraud detection field. For example, in scenarios such as credit card fraud detection, false comment identification on online shopping platforms, and spam filtering on social media, the model can quickly identify and intercept abnormal behaviors, protect user property security, and maintain platform order.

### 4.5 Comparison of computational complexity and execution time

The experimental environment uses a distributed computing cluster. Hardware configurations include 8 compute nodes equipped with NVIDIA A100 80GB Graphics Processing Units (GPUs). Each node is equipped with dual AMD EPYC 7763 processors and 1TB DDR4 memory, interconnected via an InfiniBand HDR 200Gb/s high-speed network. The software stack runs on Ubuntu 20.04 LTS using PyTorch 1.12 and DGL 0.9 frameworks. This configuration simulates real-world deployment conditions of financial-grade risk control platforms, ensuring industrial relevance for time measurement. Furthermore, the comparative results of the THG-OAFN model with other existing models (GCN, GAT, GLU-GCN) in terms of computational complexity and execution time are outlined in Table 13:

**Table 13:** Comparison of execution time (in seconds).

Model	Training time (small-scale dataset)	Training time (large-scale dataset)	Inference time (small-scale dataset)	Inference time (large-scale dataset)
GCN	120	900	5	45
GAT	150	1,100	6	55
GLU-GCN	130	950	5.5	47
THG-OAFN	200	1,300	8	60

Regarding execution time, GCN has a training time of 120 seconds on a small-scale dataset and 900 seconds on a large-scale dataset. Inference times are 5 seconds and 45 seconds, respectively. Overall, GCN performs well in terms of execution time. GAT, due to its attention mechanism, has longer training and inference times. It takes 150 seconds to train on a small-scale dataset and 1,100 seconds on a large-scale dataset. Inference times are 6 seconds and 55 seconds, respectively. GLU-GCN falls between GCN and GAT in terms of execution time. It takes 130 seconds to train on a small-scale dataset and 950 seconds on a large-scale dataset. Inference times are 5.5 seconds and 47 seconds, respectively. THG-OAFN, incorporating a time-aware mechanism, has a training time of 200 seconds on a small-scale dataset and 1,300 seconds on a large-scale dataset. Inference times are 8 seconds and 60 seconds, respectively. Despite longer execution times, the THG-OAFN model accurately captures fraudulent behavior within time series data. In terms of computational complexity, although THG-OAFN exhibits higher complexity and longer execution times, its performance improvement in fraud detection is significant. Model design must strike a balance between complexity and performance to ensure feasibility in practical applications.

To address the core requirement of millisecond-level response in financial operations, THG-OAFN’s real-time design follows a three-layer optimization strategy. At the model level, knowledge distillation is employed to compress the multi-layer attention framework into a lightweight student model, reducing parameter count by 83% while retaining 95% detection accuracy. At the architecture level, a heterogeneous computing pipeline is implemented. Central Processing Units (CPUs) preprocess time-series features, GPUs perform graph aggregation in parallel, and dual engines collaborate to reduce end-to-end latency. At the engineering level, a dynamic pruning mechanism is established to skip low-threat subgraph analysis in real time based on transaction risk probabilities. These strategies form a “model-architecture-system” optimization loop, enabling the system to meet the extreme response constraint of <200ms in high-frequency trading scenarios.

For deployment feasibility, a progressive lightweight solution is proposed. In the online service phase, quantization-aware training (QAT) converts the FP32 model to INT8 precision, with inference accelerated via the TensorRT engine. This significantly reduces computational resource overhead while maintaining detection accuracy fluctuations within <1%. During incremental updates, parameter-isolated fine-tuning is designed to unfreeze only the attention fusion layer for local updates, avoiding minute-level service interruptions caused by full-model retraining. In the disaster recovery phase, an asynchronous degradation channel is deployed to automatically switch to a hybrid mode of rule engine and lightweight GCN when transaction volume surges by 300%. This ensures system availability as a priority.

### 4.6 Generalization ability test of models

To further validate the generalization capability of the proposed model, experiments are conducted on datasets from the banking and financial sectors. Datasets in the financial domain exhibit significant differences from existing public datasets (such as Amazon, YelpChi, and Credit card fraud detection dataset), particularly in aspects like transaction behavior, user types, and fraud patterns. To test the model’s performance on such data, the following financial datasets are selected:

The bank transaction dataset: This dataset includes real bank transaction records, covering both normal and fraudulent transactions, and has a strong financial application background.

In terms of experimental setup, the same hyperparameter tuning process as described earlier is maintained, and the model is appropriately adjusted according to the characteristics of the datasets. The performance comparison between THG-OAFN and baseline models on financial datasets is shown in Table 14:

**Table 14:** Cross-domain generalization experiment: Performance comparison between THG-OAFN and baseline models on financial datasets.

Model	Dataset	Accuracy	Recall	F1-score
GCN	Bank Transaction	0.84	0.80	0.82
GAT	Bank Transaction	0.83	0.78	0.80
GRU-GCN	Bank Transaction	0.85	0.82	0.83
HGMA	Bank Transaction	0.86	0.82	0.84
THG-OAFN	Bank Transaction	0.92	0.88	0.90
GCN	Credit Card Fraud	0.75	0.52	0.47
GAT	Credit Card Fraud	0.77	0.59	0.52
GRU-GCN	Credit Card Fraud	0.80	0.69	0.62
HGMA	Credit Card Fraud	0.87	0.84	0.82
THG-OAFN	Credit Card Fraud	0.93	0.91	0.92

Table 14 indicates that THG-OAFN achieves an F1-score of 0.90 on the bank transaction dataset, significantly outperforming the baseline model (HGMA: 0.84), with a recall of 0.88, indicating the ability to capture 1,683 fraudulent transactions. On the credit card fraud dataset, THG-OAFN maintains a stable F1-score of 0.92 (10 percentage points higher than HGMA), and a recall of 91% verifies its sensitivity to 492 extreme minority-class samples. Notably, GRU-GCN’s recall fluctuates by 13 percentage points between bank transactions (0.82) and credit card scenarios (0.69), exposing temporal models’ sensitivity to transaction behavior differences. Bank transactions exhibit stronger periodic patterns (e.g., salary disbursements, bill payments), while credit card fraud involves more sudden account takeovers. THG-OAFN maintains an F1-score>0.90 across both financial datasets, demonstrating domain adaptability through heterogeneous graph abstraction and dynamic oversampling. Specifically, in bank data, the model successfully identifies the pattern of “high-frequency small-amount test transactions followed by large transfers,” reducing false positive rates.

Performance comparisons for insurance claim fraud detection are suggested in Table 15. THG-OAFN demonstrates exceptional generalization abilities across three major insurance domains. In the medical insurance dataset, it identifies the “false hospitalization diagnostic code” fraud pattern with an F1-score of 87.4%, outperforming the best baseline FraudGNN-RL by 5.8 percentage points. In property insurance scenarios, it successfully detects two core patterns—”duplicate claims” (multiple filings for the same loss) and “loss exaggeration”; an F1-score achieves 84.9%, primarily due to the dynamic weighting of “adjuster-supplier-customer” triangular relationships by the relationship fusion module. The F1-score of 89.2% in the auto insurance claim dataset confirms the model’s sensitivity to sudden temporal patterns, such as capturing the “post-accident supplementary insurance” fraud chain. Migration learning experiments further validate adaptability. Using the Amazon-pretrained THG-OAFN as a foundation, fine-tuning only the attention fusion layer (50 epochs) on medical insurance data achieves an F1-score of 85.7%, demonstrating effective cross-domain knowledge transfer.

**Table 15:** Performance comparison of insurance claim fraud detection (F1-score/%).

Model	The medical insurance dataset	The property insurance dataset	The auto insurance claim dataset	Average improvement
GCN	72.3	68.5	75.1	–
GAT	74.8	70.2	76.9	–
FraudGNN-RL	81.6	77.3	83.4	–
**THG-OAFN**	**87.4**	**84.9**	**89.2**	**+6.8**

### 4.7 Discussion on engineering deployment and reproducibility

To enhance the real-world deployment ability of the proposed THG-OAFN model, this section discusses its engineering deployment plan and reproducible implementation path in large-scale EC fraud detection systems.

Regarding deployment architecture, addressing the high concurrency and strong timeliness requirements of transaction behaviors, THG-OAFN can be implemented as a distributed detection system based on cloud platforms (e.g., AWS or Alibaba Cloud). The model is encapsulated as a standard service interface, enabling high-concurrency calls via RESTful APIs to connect with business middle platforms. The data collection and preprocessing module constructs real-time transaction data streams through Kafka message queues and Flink stream processing frameworks. Meanwhile, heterogeneous graph slices are automatically generated by a time window for input into the detection module. The model itself runs in a GPU-accelerated container environment (e.g., Kubernetes + TensorRT) to achieve low-latency response and dynamic scaling management.

To address performance challenges from large-scale graph computing, lightweight graph computing frameworks such as Deep Graph Library (DGL) or PyTorch Geometric (PyG) are introduced in engineering implementations. At the same time, GraphSAGE-style neighbor sampling is used to avoid memory bottlenecks from full-graph expansion. During inference, the model is optimized via knowledge distillation, pruning, and quantization compression to transform complex multi-layer attention structures into lightweight sub-models while preserving core representation capabilities. Additionally, an incremental learning mechanism employs state caching and gradient freezing strategies to enable fast fine-tuning updates as new data arrives, reducing retraining overhead and enhancing system robustness.

To improve reproducibility and system reliability, the development process adheres to modular design principles, packaging data processing, graph construction, feature extraction, attention calculation, classifier training, and deployment as standardized components. Yet Another Markup Language (YAML) configurations are used for unified hyperparameter tuning, combined with version control and log tracking to ensure stable and reproducible model results. Plans include open-sourcing partial modules to facilitate community reproducibility and migration.

Despite the proposed model’s high accuracy and generalization in structural design and experimental validation, several challenges remain for practical engineering implementation. First, graph structure storage and computational resource consumption remain significant when handling millions of nodes and multi-type relationships. This necessitates future exploration of graph partitioning and caching-based distributed training strategies. Second, the current model lacks systematic regularization control, carrying a risk of overfitting, particularly in data segments with drastic changes in fraudulent behaviors. Subsequent optimizations may introduce graph contrastive learning, DropEdge, or other regularization strategies. Third, while incremental learning reduces latency to some extent, it still falls short of millisecond-level real-time detection requirements. Future research may focus on low-latency solutions based on edge deployment and asynchronous model inference.

### 4.8 Comprehensive discussion

To sum up, this study proposes an innovative THG-OAFN detection method aimed at the increasingly serious problem of Internet fraud in the EC domain. This method makes core contributions in the following aspects and effectively solves the research problems proposed in the introduction: (1) Time awareness ability: By integrating GRU, the THG-OAFN model effectively captures the time series characteristics of transaction data, thus overcoming the shortcomings of traditional methods in processing time dynamics. (2) Heterogeneous graph data abstraction: The model utilizes heterogeneous GNN to process static features in transaction data, and abstracts the relationship between users and transactions employing heterogeneous graphs, thereby enhancing the expression ability of complex transaction patterns. (3) Data imbalance solution: By introducing a framework based on oversampling, especially the SMOTE, the model effectively handles the problem of class imbalance in fraud detection and improves the identification accuracy of minority classes (fraudulent transactions). (4) Introduction of attention mechanism: The model employs an attention mechanism to quantify the importance between nodes and enhance the ability to identify key features in transaction data, to predict fraud more accurately. (5) The multilayer attention-based heterogeneous graph fraud detection framework: By constructing a multilayer framework including neighborhood fusion, relationship fusion, and information perception modules, the model can actively identify and predict fraud patterns, realizing the transformation from passive detection to active prevention. (6) Experimental verification: The experimental results on Amazon and YelpChi public datasets denote that the THG-OAFN model outperforms the existing GNN model in key evaluation metrics such as AUC, Recall, and F1-score, which proves its effectiveness in dynamic fraud detection and active fraud prevention.

The advantages of the proposed THG-OAFN model enable it to seamlessly integrate into practical fraud detection systems in the following ways:

1) System integration: The proposed model can be deployed as part of existing fraud detection pipelines. It can process transaction data in real-time, providing scores or labels for suspicious transactions. This real-time processing capability is crucial for quickly identifying and responding to fraudulent activities. 2) Application scenarios: Potential applications include credit card fraud detection, online banking fraud prevention, and e-commerce fraud prevention. The model’s ability to handle temporal and heterogeneous data makes it suitable for various fraud scenarios. Its flexibility allows it to adapt to different industry needs. 3) Performance evaluation: In real-world environments, the model’s performance can be continuously assessed and updated through feedback loops. Metrics such as detection accuracy, false positive rate, and response time are essential for evaluating the model’s effectiveness and making necessary adjustments. Continuous monitoring and optimization ensure that the model remains efficient amid evolving fraud patterns.

However, the experimental process also comes with some unknown risks. For example, when using oversampling techniques to address data imbalance, there is a potential risk of overfitting, especially when the oversampled minority class causes the model to memorize the training data rather than generalize. To mitigate this risk, the following measures are taken. Cross-validation: K-fold cross-validation is employed to ensure the model’s consistent performance across different data subsets, thus reducing the likelihood of overfitting. Cross-validation helps assess the model’s stability and generalization ability. Regularization: L2 regularization is applied to prevent the model from becoming overly complex, thereby avoiding fitting noise in the training data. Regularization controls the complexity of the model by adding a penalty term to the loss function. Ensemble methods: Multiple models are combined into an ensemble to reduce overfitting risk by decreasing the variance component of model errors. Ensemble methods enhance the model’s stability and robustness. By implementing these measures, while learning from oversampled data, the model ensures good generalization capability, thereby mitigating the risk of overfitting.

Another example is the high computational complexity and memory requirements of the THG-OAFN model, which may limit its practical application. Therefore, future optimization will also focus on reducing these computational and memory demands. For instance, parallel computing: Multi-threading or GPU acceleration reduce training and inference times. Model pruning: Pruning techniques mitigate model parameters and computational complexity. Efficient data structures: More efficient data structures and algorithms are adopted to optimize graph construction and feature extraction processes. Through this analysis, the characteristics of the THG-OAFN model in terms of computational complexity and execution time are demonstrated, further validating its potential and challenges in practical applications. Consequently, continued model optimization is necessary to enhance its efficiency and performance in large-scale data processing.

Additionally, in real-world applications, fraud patterns often change. To enable real-time detection of new fraudulent behaviors, the proposed model also needs to possess online learning and incremental learning capabilities. In the experiments, scenarios with incremental data are simulated, gradually adding new fraudulent data to the training set, and evaluating how the model’s performance changes during this process. The results indicate that the THG-OAFN model effectively adapts to the addition of new data while maintaining high detection accuracy. Furthermore, after incorporating new data, the model undergoes parameter fine-tuning to adjust to the new data distribution. Multiple experiments simulate scenarios with different data arrival rates and changes in fraud patterns. The experimental results demonstrate that the THG-OAFN model can quickly adapt to new data during incremental learning processes while maintaining high detection performance.

## 5. Conclusions

The THG-OAFN framework proposed in this study achieves methodological innovations in three aspects: temporal-aware heterogeneous graph oversampling, hierarchical attention fusion mechanism, and active fraud detection strategy. First, the study proposes an oversampling strategy that introduces topological constraints in the embedding space. This strategy significantly alleviates the problems of scarcity of fraud samples and class imbalance, and enhances the model’s ability to learn minority class behaviors. Second, it designs a relationship-hierarchy-multi-head progressive attention system. This system aims to mine high-order potential fraud patterns in multi-relationship transaction networks, improving the model’s expression ability for complex graph structures. Third, it introduces an incremental feedback mechanism, enabling the model to dynamically update the knowledge base during detection and realize the transformation from passive discrimination to active early warning. Experimental results show that on two public datasets, THG-OAFN maintains leading detection performance (AUC of 96.56%, recall of 95.21%, and F1 score of 94.72% on the Amazon dataset). Meanwhile, it remarkably reduces the average false alarm rate, demonstrating robustness in high-dimensional, dynamic, and imbalanced scenarios.

### 5.1 Main contributions of the model

This study forms the following technical closed-loop in the method structure design. (1) Oversampling mechanism constructs topological consistency constraints based on the embedding space, generates new fraud nodes and edge structures, retains semantic consistency, and performs attribute completion through HGNNAC; (2) Multi-layer attention mechanism includes three modules of relationship fusion, neighbor fusion, and information perception, extracts different levels of information in the graph layer by layer, and introduces a multi-head mechanism to stabilize learning; (3) Active fraud detection mechanism dynamically captures the evolution path of transaction behaviors through GRU-GNN fusion, supplemented by rolling window and knowledge base feedback mechanisms to improve early warning capabilities and model adaptability.

### 5.2 Analysis of model limitations and challenges

Although the proposed THG-OAFN model achieves relatively significant results in both structural design and experimental verification, it still has three main limitations and potential failure risks. (1) Computational and storage overhead: As the number of nodes and types of relationships expand, the hierarchical attention mechanism (especially multi-head attention) causes memory and computational load to grow superlinearly. This limits the model’s ability for real-time deployment on ultra-large-scale transaction graphs. Future optimization is needed using lightweight methods such as model pruning and parameter sharing. (2) Response latency bottleneck: The current window-level incremental training improves model adaptability, but it still cannot meet sub-second response requirements in high-frequency streaming data scenarios. Future consideration can be given to adopting GPU-CPU pipeline scheduling and graph operator parallel mechanisms to reduce inference time. (3) Privacy compliance issues: At this stage, the model assumes a centralized data storage environment and does not fully address the privacy protection needs of cross-platform transaction data. Subsequent efforts should introduce federated learning and differential privacy mechanisms to avoid data leakage and ensure the security of model weights.

In addition, THG-OAFN may face the risk of performance degradation in the following extreme scenarios. (1) When fraud patterns evolve swiftly: If fraud strategies undergo drastic changes in a short period, historical data may fail to effectively guide current detection, leading to “concept drift.” (2) When the graph structure is severely imbalanced: If connections between fraud nodes are sparse, newly generated nodes through oversampling may struggle to construct effective topological contexts, affecting detection results. (3) Under extremely low-resource conditions: When available labeled samples are extremely few, the model may fall into local optima due to improper initialization, impairing generalization ability.

### 5.3 Prospects for subsequent research directions

To address the above issues, future research can focus on the following three technical directions. (1) Spatiotemporal coupled dynamic graph modeling: Continuous-time point processes combine with graph convolution methods to uniformly encode transaction time, amount, and structural evolution features, enhancing the model’s ability to capture sudden and latent fraud behaviors. (2) Lightweight deployment and accelerated inference: A lightweight inference framework is constructed through model pruning, parameter sharing, graph operator parallelization, and other means. Moreover, end-to-end latency is optimized by combining heterogeneous hardware scheduling mechanisms. (3) Privacy protection and federated collaborative learning: Differential privacy perturbation and secure multi-party computation protocols are introduced to ensure the confidentiality of transaction data in cross-platform collaborative detection and prevent model weights from being reverse-engineered.

The THG-OAFN framework proposed in this study demonstrates innovation and practicality in theory and practice. Through the above supplementary content, it further strengthens the systematic analysis of contributions, structure, limitations, and challenges, providing a solid foundation for future research on intelligent fraud prevention and control.

## Supporting information

S1 File THG-OAFN source code and experimental scripts. This compressed archive contains the implementation of the Temporal-aware Heterogeneous Graph Oversampling and Attention Fusion Network (THG-OAFN) model together with the scripts required to reproduce the experiments reported in this study. The package includes source code for model construction, data preprocessing, training and evaluation pipelines, as well as configuration files and a README document that provides basic instructions for running the code.
(ZIP)
